# Measuring human capital using global learning data

**DOI:** 10.1038/s41586-021-03323-7

**Published:** 2021-03-10

**Authors:** Noam Angrist, Simeon Djankov, Pinelopi K. Goldberg, Harry A. Patrinos

**Affiliations:** 1 World Bank, Washington, DC USA; 2 Department of Economics and Blavatnik School of Government, University of Oxford, Oxford, UK; 3 Financial Markets Group, London School of Economics, London, UK; 4 Peterson Institute for International Economics, Washington, DC USA; 5 Department of Economics, Yale University, New Haven, CT USA; 6 Bureau for Research and Economic Analysis of Development (BREAD), Kennedy School of Government, Harvard University, Cambridge, MA USA; 7 Centre for Economic Policy Research (CEPR), London, UK; 8 National Bureau of Economic Research (NBER), Cambridge, MA USA

**Keywords:** Developing world, Economics, Education

## Abstract

Human capital—that is, resources associated with the knowledge and skills of individuals—is a critical component of economic development^1,2^. Learning metrics that are comparable for countries globally are necessary to understand and track the formation of human capital. The increasing use of international achievement tests is an important step in this direction^3^. However, such tests are administered primarily in developed countries^4^, limiting our ability to analyse learning patterns in developing countries that may have the most to gain from the formation of human capital. Here we bridge this gap by constructing a globally comparable database of 164 countries from 2000 to 2017. The data represent 98% of the global population and developing economies comprise two-thirds of the included countries. Using this dataset, we show that global progress in learning—a priority Sustainable Development Goal—has been limited, despite increasing enrolment in primary and secondary education. Using an accounting exercise that includes a direct measure of schooling quality, we estimate that the role of human capital in explaining income differences across countries ranges from a fifth to half; this result has an intermediate position in the wide range of estimates provided in earlier papers in the literature^5–13^. Moreover, we show that average estimates mask considerable heterogeneity associated with income grouping across countries and regions. This heterogeneity highlights the importance of including countries at various stages of economic development when analysing the role of human capital in economic development. Finally, we show that our database provides a measure of human capital that is more closely associated with economic growth than current measures that are included in the Penn world tables version 9.0^14^ and the human development index of the United Nations^15^.

## Abstract (web summary)

Analyses of a global database reveal that in many developing countries progress in learning remains limited despite increasing enrolment in primary and secondary education, and uncover links between human capital and economic development.

## Main

The notion of human capital was mentioned as early as in 1776^16^ and formalized two centuries later^17^. Ever since, researchers have explored the role of human capital in economic development. For decades, studies used measures of schooling as a proxy for human capital^18–20^. This applies even to the most prominent index of human capital to date, the United Nation’s human development index (HDI).

However, using schooling as a proxy for human capital assumes that being in school translates to learning. Evidence suggests that this is often not the case^21^. A recent analysis reveals that six out of ten adolescent individuals worldwide cannot meet basic proficiency levels in mathematics and reading^22^. The gap between schooling and learning is acute in developing countries. In Kenya, Tanzania and Uganda, three-quarters of the students in grade 3 cannot read a basic sentence such as ‘the name of the dog is Puppy.’^1^. In rural India, half of the students in grade 3 cannot solve a two-digit subtraction problem (such as 46 – 17)^1^.

These data from previous studies demonstrate a substantial gap in the formation of human capital: students are in school, but do not learn enough. Closing this gap is an important priority for economic development. Several studies have suggested that when human capital is measured by schooling, it does not deliver the returns predicted by growth models. However, when measured by learning, human capital is more strongly associated with growth^3,23,24^.

To date, much of the effort to measure learning has focused on high-income countries. This limitation is due to the absence of comparable measures of learning in low- and middle-income countries. Existing measures exclude a considerable portion of the global distribution, in particular countries with the most potential to gain from the accumulation of human capital.

In this Article we bridge this gap. We introduce a database of globally comparable learning outcomes for 164 countries covering 98% of the global population from 2000 to 2017. This is one of the largest and most-current global learning databases, one of the first to disaggregate learning results by gender and to introduce methodological improvements such as the inclusion of standard errors to quantify uncertainty around mean scores. The database, referred to as the Harmonized Learning Outcomes (HLO) database, is available for public use and updates are expected every 2 to 3 years as new learning data become available (see ‘Data availability’in Methods). A large-scale effort to track the formation of human capital using this database is the World Bank’s new human capital index^25^.

Of note, throughout this Article, we use the term ‘schooling’ when referring to the average years of schooling or average enrolment rates of a country at specific schooling levels. We use the term ‘learning’ when referring to the stock of basic cognitive skills, including mathematics, reading and science, as measured by standardized tests conducted in school.

## HLO database

The database was produced through a large-scale effort by the World Bank to identify, collect and collate student assessment data worldwide. We include seven assessment regimes in total: three international tests, three regional standardized achievement tests and the Early Grade Reading Assessment, which adds 48 countries to the database with at least one data point in the past 10 years, including large developing economies such as Bangladesh, Nigeria and Pakistan. Each test covers between 10 and 72 countries. By combining these assessments and making them comparable, we include countries that represent 98% of the global population. A detailed description of the methodology that we use to develop harmonized learning measures as well as all data included in the database are provided in the Methods and Supplementary Information II.

The database includes mean scores as well as standard errors for each measure, in an attempt to quantify uncertainty. Scores are disaggregated by schooling level (primary and secondary), subject (reading, mathematics and science) and gender (male and female). We include year-by-year data. We do not extend the time series before 2000 as the quality of the data is low for the period before 2000.

The coverage and detail of the database is described further in Extended Data Table 1 and the Supplementary Information. The database includes 2,023 country–year observations from 2000 to 2017 (Extended Data Table 1). Disaggregation by gender is available for 98.5% of observations. Latin America and the Caribbean and sub-Saharan Africa make up 21% of all available data. Additional descriptive statistics are provided in Supplementary Information IA.

Our methodology uses the expansion of international assessments to construct globally comparable learning outcomes. These tests are derived from assessments conducted in the USA since the 1960s, such as the Scholastic Achievement Tests (SATs) and the National Assessment of Educational Progress (NAEP). The tests are psychometrically designed, standardized assessments of cognitive skills. Since the 1990s, international assessments have been conducted by organizations such as the The Organisation for Economic Co-operation and Development (OECD). Two high-profile examples are the Programme for International Student Assessment (PISA) and the Trends in International Mathematics and Science Study (TIMSS), which covered 71 and 65 countries, respectively, in 2015. These assessments enable credible global comparisons of learning across countries and over time. However, to date most analyses of these assessments cover few developing countries^3,26–29^.

We include 164 countries, two-thirds of which are developing countries, by linking international assessments to their regional counterparts. Regional assessments cover much of sub-Saharan Africa and Latin America but have often been excluded from international comparisons. We convert a regional test score to an international test score within subjects (mathematics, reading and science) and schooling levels (primary and secondary) and within adjacent years. By including tests across the same testing round and at the disaggregated schooling and subject level, this method minimizes the likelihood that test differences are a function of time, proficiency, schooling level or data availability. We then apply this conversion to a country that participates in a regional test but not an international test to produce a comparable score (referred to as a HLO in the database). Mean scores are also calculated for disaggregated groups—for example, scores were calculated for each gender. The detailed methodology is described in the Methods and Supplementary Information II.

By constructing a conversion method across tests between international and regional assessments, we quantify the difference between tests, adjust for this difference and place learning outcomes from regional assessments on a global scale. For a high-performance benchmark, we use the TIMSS benchmark of 625. For the low-performance benchmark, we use 300, which is the equivalent on the HLO scale of the minimum benchmarks for regional assessments such as The Laboratorio Latinoamericano de Evaluación de la Calidad de la Educación (Latin-American Laboratory for Assessment of the Quality of Education (LLECE)) and The Programme for the Analysis of Education Systems (PASEC). This approach enables us to capture performance across the distribution of both international and regional benchmarks.

Data harmonization efforts such as the one described in this Article serve the dual purpose of compiling the best available data at a given point in time and motivating additional data collection. Thus, they set in motion a cycle that can continually improve learning data over time. For example, in the most recent release of the World Bank human capital index, 20 new countries participated in learning assessments for the first time, enabling their inclusion in subsequent versions of this database.

## Schooling is not learning

**Fig. 1:**
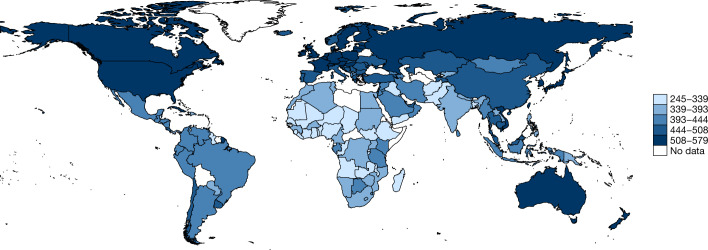
Average learning (2000–2017). Learning scores are calculated from our HLO database as averages for a given country across subjects and levels over the time period 2000–2017. The numbers in the legend are the lowest and highest average HLO learning scores when averaged over the time period 2000–2017. Average scores by region are as follows: East Asia and Pacific (445), Europe and Central Asia (489), Latin America and the Caribbean (402), Middle East and North Africa (399), North America (529), sub-Saharan Africa (342) and South Asia (335). A few trends emerge: sub-Saharan African regions lag behind all regions apart from South Asia, with countries such as India (368) performing on par with lower-performing sub-Saharan African countries such as Uganda (369); within sub-Saharan Africa, a few countries such as Kenya (444) and Tanzania (416) lead, on par with many countries in Latin America such as Mexico (435); within Latin America, a few countries such as Chile (449) lead compared with some European counterparts such as Georgia (437); the Middle East performs similarly or worse than Latin America (as shown in summarized scores by region); many Asian countries outperform North American and European regions (for example, Japan (553) relative to the United States (521)).

We present a few descriptive trends in a first application of the database. The average learning outcomes for 164 countries from 2000 to 2017 is shown in Fig. 1. The global coverage of the database becomes immediately apparent and regions typically excluded from international tests such as PISA and TIMSS included in our database are clearly shown (Fig. 1). The database covers the vast majority of countries in sub-Saharan Africa, Latin America and the Caribbean, and South Asia— economies with considerable potential to close learning gaps.

A few trends emerge: high-income countries far outpace developing economies. Sub-Saharan African lags behind all regions besides South Asia, with countries such as India performing similar to lower-performing sub-Saharan African countries; within sub-Saharan Africa, a few countries such as Kenya and Tanzania lead, on par with many countries in Latin America. Within Latin America, a few countries such as Chile are on par with European countries. The Middle East performs similarly or worse than Latin America and many Asian countries outperform North American and European countries.

**Extended Data Fig. 1:**
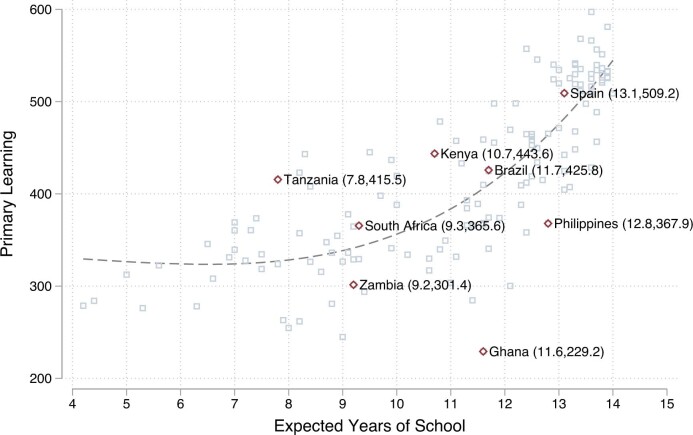
Expected years of schooling versus learning basic skills. We compare the average country learning outcomes in the latest available year from our database to the latest expected years of schooling in a given country. For example, in Zambia (9.2, 301.4) the average child can expect to receive 9.2 years of schooling and achieve learning outcomes of 301.4. The data in this figure include expected years of schooling from the World Bank Human Capital Index based on data compiled by UNESCO^25^. Primary learning outcomes are from our database. Both measures take the data in the latest year available.

The expected years of schooling and HLO primary learning scores for the most recent year for which data are available are shown in Extended Data Fig. 1 to contrast the quantity and quality of the education system (detailed descriptions of the variables and analyses of the trends are provided in the Methods). The graph shown in Extended Data Fig. 1 shows that although many developing countries have achieved substantial levels of schooling (a typical education system is expected to deliver 10–12 years of schooling), they have not yet realized high rates of learning (advanced proficiency in international tests is centred around a score of 625). Two examples with high schooling but low learning are Brazil and Ghana. Brazil has 11.7 years of expected schooling, yet a learning score of just 426. Ghana has 11.6 years of expected schooling, yet a learning score of only 229.

We next explore the contrast between changes in schooling and changes in learning over time. We measure schooling using adjusted enrolment ratios^29^. We compare this measure of schooling to our measure of learning in primary school for the years 2000–2015. We use data for this period as it has the highest overlap of schooling and learning measures. We restrict our comparison to countries with data points in at least two time periods for enrolment and learning in primary school to maximize comparability over the time period. We further condition on country-fixed effects using multivariate regression for each region (see Methods for details). This accounts for potential changes in the sample composition of countries with available data for each time period.

**Extended Data Fig. 2:**
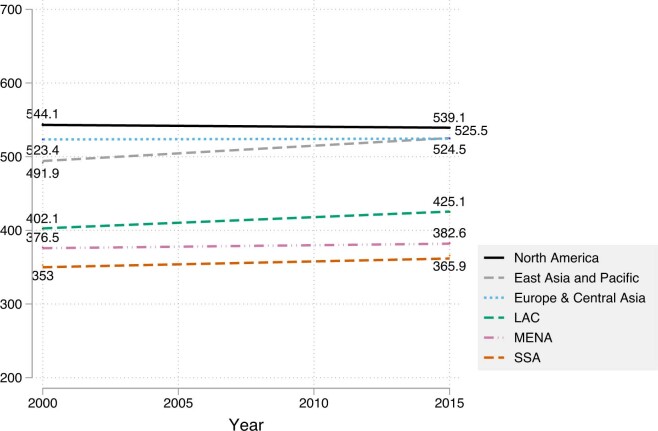
Learning in primary school by region, conditional on country-fixed effects and primary enrolment rates. We have 72 countries with both learning and enrolment data in primary school with at least two data points over the time period (2000–2015). Estimates are calculated controlling for country-fixed effects using multivariate regression by region. For each region *r* and outcome *y*, we run the following specification: ${y}_{ct}^{r}={\alpha }^{r}+{\beta }^{r}t+{{\boldsymbol{\delta }}}_{c}^{r}+{\gamma }_{ct}^{r}+{\varepsilon }_{ct}^{r}$, in which *t* represents the year, **δ** represents a vector of dummy variables for each country *c* in a given region *r* and *γ* represents primary enrolment rates. We then recover regional time trends accounting for country-fixed effects and conditional on enrolment. The data in this figure includes primary enrolment rates. This measure captures the ratio of all individuals enrolled in a given level of schooling by the population of the age group that should be enrolled at that level according to national regulations or customs accounting for grade repetition. This measure has frequently been used in the literature^29,32^. Learning estimates are taken from our database. LAC, Latin American and the Caribbean; MENA, Middle East and North Africa; SSA, sub-Saharan Africa.

We observe a clear trend towards increased schooling, while learning progress appears to be limited in many cases. For example, in the Middle East and North Africa enrolment rates achieved a high of 99% by 2010, increasing from 95% in 2000. By contrast, learning levels stayed low and remained the same around an initial score of 380 from 2000 to 2015 in these regions. It is possible that in regions such as sub-Saharan Africa, as enrolment gets substantially higher and new, lower-performing students participate in learning assessments, average scores decrease owing to a selection effect. However, we observe slow learning progress even in regions in which enrolment levels are relatively constant and high, such as Latin America and the Caribbean, suggesting that there is more to these trends than selection. In Extended Data Fig. 2, we explicitly condition on enrolment and find nearly identical patterns. Moreover, a regression of primary learning outcomes on primary enrolment rates using a panel of countries between 2000 and 2015 with country-fixed effects yields a negative coefficient on enrolment rates of 0.247 with a *P* value of 0.673, further showing that higher levels of school enrolment has no statistically significant association with better learning outcomes (see Methods for details).

**Fig. 2:**
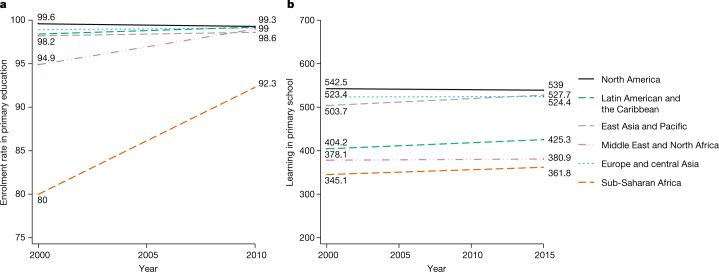
Enrolment versus learning by region, conditional on country-fixed effects. **a**, **b**, We have 72 countries with data in primary school with at least two data points for the analysed time period (2000–2015) for enrolment (**a**) and learning (**b**). Estimates are calculated controlling for country-fixed effects using multivariate regression by region. For each region and outcome *y* (primary enrolment and learning), we estimate the following specification: ${y}_{ct}^{r}=\,{\alpha }^{r}+{\beta }^{r}t+{{\boldsymbol{\delta }}}_{c}^{r}+{\varepsilon }_{ct}^{r}\,$where *t* represents the year and **δ** represents a vector of dummy variables for each country *c* in a given region *r*. On the basis of this regression, we recover regional time trends accounting for country-fixed effects. The data in this figure include primary enrolment rates. This measure captures the ratio of all individuals enrolled in a given level of schooling to the population of the age group that should be enrolled at that level according to national regulations or customs accounting for grade repetition. This measure has frequently been used in the literature^26,29,32^. Learning estimates are taken from our database.

**Extended Data Fig. 3:**
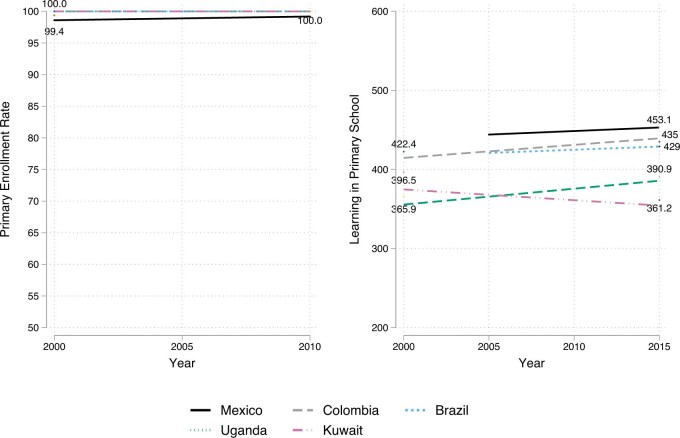
Learning by country. Left, primary enrolment rates data are from a previous study^29^. Right, learning estimates are derived from our database and are plotted using a linear fit for the available data over the time series. Data on learning are available across the time period for Uganda from the source test the Southern African Consortium for Monitoring Education Quality (SACMEQ). For Kuwait, data are from The Progress in International Reading Literacy Study (PIRLS) and TIMSS. Data are available for Brazil, Mexico and Colombia from the LLECE as well as PIRLS for Colombia.

The patterns in Fig. 2 and Extended Data Fig. 2 could be interpreted as indicative of a plateau effect, as at higher learning levels—for example, in North America or Europe—obtaining further gains may be difficult. However, we also see a relatively flat line in cases in which baseline learning levels are low—that is, in Latin America and the Caribbean—which suggests that learning progress is slow regardless of initial learning conditions. Data availability for each country could in principle affect the patterns that we describe, but the robustness of the patterns to the inclusion of country-fixed effects as described above suggests that they are not driven by country composition. Country-specific trends are also illustrated in Extended Data Fig. 3.

Taken together, the data reveal that the rapid pace at which schooling improved across education systems, as well as the success in achieving high enrolment rates, have not been met with similarly rapid learning progress. This pattern has been referred to in the literature^1^ and by the international education community as ‘the learning crisis’^1, 22^. Using the HLO database, we demonstrate that it holds on a global scale.

## Human capital and economic development

A large number of studies in the development accounting literature have explored the relative contribution of human capital to cross-country income differences. However, the results have been inconclusive, in part owing to difficulties in measuring human capital. Although direct measures of years of schooling exist, the quality of schooling has been harder to measure.

Approaches to estimate the quality of schooling across countries have relied on differences in Mincerian wage returns^5,6^, immigrant returns^7^ and cross-country skill premia^8^. However, these approaches face several challenges, including the need to make assumptions about the substitutability of skilled and unskilled workers^9^. The challenges in measuring quality have contributed to substantial variation in estimates of the role of human capital in accounting for cross-country differences in income, ranging from nearly all to potentially none^5–13^.

In this study, we provide a more direct and reliable measure of the quality of schooling based on our learning outcome data, which we use to construct measures of human capital stock (Methods).

Our results (Table 1) suggest that human capital accounts for between a fifth to around half of cross-country differences in income—which is an intermediate position relative to the estimates found in the literature^5–13^, which range from zero to nearly all. These results are consistent with models of human capital that capture the role of educated entrepreneurs and more comprehensive measures of human capital that include schooling, learning and health^30,31^.

**Table 1:** Baseline development accounting results and comparison to the literature

Human capital contribution	Our estimates				Estimates from the literature			
	*w* = 0	*w* = 0.15	*w* = 0.20	*w* = 0.25	Ref. ^5^	Ref. ^12^	Ref. ^7^	
*h*_90_/*h*_10_	2.24	3.35	3.82	4.34	2.00	2.10	4.70	
(*h*_90_/*h*_10_)/(*y*_90_/*y*_10_)	0.11	0.16	0.19	0.21	0.09	0.22	0.21	
var(log[*h*])/var(log[*y*])	0.07	0.14	0.18	0.21	0.06	0.07	0.26	
	**w = 0**	**w = 0.15**	**w = 0.20**	**w = 0.25**	**Ref.**^10^	**Ref.**^44^	**Ref.**^13^	**Ref.**^11^
(ln[*h*_90_] – ln[*h*_10_])/(ln[*y*_90_] – ln[*y*_10_])	0.27	0.40	0.44	0.48	Nearly all	0.51	0.62	Potentially none

The variable *y* is real output per worker on average from 2000 to 2010; *h* is a measure of human capital constructed on the basis of both schooling and learning data on average from 2000 onwards. We include various decompositions of the role of human capital in explaining cross-country income differences based on the literature^5–13^: *h*_90_/*h*_10_, (*h*_90_/*h*_10_)/(*y*_90_/*y*_10_), var(log[*h*])/var(log[*y*]), (ln[*h*_90_] – ln[*h*_10_])/(ln[*y*_90_] – ln[*y*_10_]). Subscripts refer to percentiles; for example, the decomposition (*h*_90_/*h*_10_)/(*y*_90_/*y*_10_) captures the ratio of human capital in the 90th relative to 10th percentile over the real output per worker in the 90th relative to 10th percentile. Variable constructions and decompositions are described in detail in the Methods. We assume rates of return to the learning component of human capital—denoted as *w*—on the basis of the microeconomic literature^42,43^. We conduct sensitivity analyses with values *w* = 0.15, w = 0.20, and *w* = 0.25. When *w* = 0, our accounting de facto only includes schooling; for any value *w* > 0, we include learning as well as schooling. We include 131 countries in this development accounting exercise. Schooling data are from a previously published study^32^. GDP data on real output per worker are from the Penn world tables v.9.0^14^. Learning estimates are from our database. Literature estimates are derived from previously published papers^5–13,44^.

The average relationship between learning and income masks significant heterogeneity across countries (Extended Data Table 2). We find that human capital explains less than two-fifths of cross-country income differences among low-income countries, but more than half among high-income countries. We find even larger differences across regions. For example, when measured by schooling, human capital accounts for 54% of cross-country income differences in advanced economies and only 4% in sub-Saharan Africa. When we include learning, this gap widens to 86% in advanced economies but only 10% in sub-Saharan Africa. This substantial heterogeneity reveals the importance of including a global distribution of countries covering multiple stages of economic development when examining the role of human capital.

Finally, we compare our measure of human capital to alternative measures that are based on prominent global databases such as the Penn world tables^14^, the Barro–Lee Educational Attainment dataset^32^ and the United Nation’s HDI^15^. In Table 2, we find that our measure of human capital has a stronger association with growth than alternative human capital measures. This is the case in univariate regressions that include each measure on its own (columns 1–4). We observe that a change of 1% in learning is associated with a change of 7.2% in annual growth. By contrast, a change of 1% in the other human capital measures is associated with a change of between 1.6% and 3.3% in annual growth. We further show that when we include all measures in the same multivariate regression, the association between our measure of learning and growth remains high, between 5.9% and 6.9%, and statistically significant (*P* ≤ 0.01), whereas other human capital variables have a reduced and statistically nonsignificant association with growth. We find that the model fit improves only slightly when all measures are included with an *R*^2^ value of 0.32 relative to our measure of human capital with an *R*^2^ value of 0.30.

**Table 2:** Comparing measures of human capital and economic growth

	Annual growth rate (2000–2010)							
	1	2	3	4	5	6	7	8
Human capital (harmonized learning outcomes)								
Regression coefficient	0.072				0.059	0.061	0.069	0.066
Standard error	0.018				0.023	0.022	0.023	0.024
*P* value	0.000				0.011	0.007	0.003	0.006
Human capital (Penn world tables)								
Regression coefficient		0.033			0.012			0.019
Standard error		0.011			0.013			0.035
*P* value		0.003			0.358			0.597
Human capital (schooling^32^)								
Regression coefficient			0.016			0.006		0.014
Standard error			0.006			0.007		0.020
*P* value			0.004			0.382		0.494
Human capital (HDI)								
Regression coefficient				0.020			0.002	−0.028
Standard error				0.008			0.010	0.022
*P* value				0.013			0.844	0.914
Control for GDP at the start of the time period	Yes	Yes	Yes	Yes	Yes	Yes	Yes	Yes
Observations	107	107	107	107	107	107	107	107
*R*^2^	0.300	0.261	0.255	0.240	0.306	0.305	0.301	0.318

Columns 1–8 include the same dependent variable. The difference across columns depends on which independent variables are included as denoted by whether the rows are filled out. The dependent variable is the annual growth rate averaged across 2000–2010^14^. Human capital (harmonized learning outcomes) refers to the measure of human capital in this database averaged from 2000 onwards. Human capital (Penn world tables) refers to the measure of human capital in the Penn world tables v.9.0^14^. Human capital (schooling^32^) refers to estimates from 2000, which is the start of our sample period^32^. Human capital (HDI) refers to the measure of education included in the United Nation’s HDI in the year 2000^15^. Results include 107 countries and exclude countries in civil war, inflation crises and with rents from natural resources above 15%. All independent variables are transformed to log units to derive comparable elasticities. We control for the log transformation of initial GDP per capita levels at the beginning of the period (in the year 2000) in all specifications following standard practice in the growth literature^3^. We report regression coefficients, standard errors of the regression and *P* values.

Therefore, our measure of human capital appears to have a stronger relationship with economic growth, both individually and jointly. This is probably because alternative measures of human capital rely largely on years of schooling and omit learning. However, the use of these alternative measures remains standard practice, in part because these data have the broadest coverage. By constructing learning data across 164 countries, we fill a key gap: broad coverage over nearly two decades and a measure of human capital with strong links to economic development.

## Discussion and future directions

This database comes at a moment when a series of global efforts have been launched to measure and track learning on a global scale. Although recent modelling suggests that the world is on track to achieve the goal of universal primary enrolment by 2030^33^, if learning continues to stagnate, this achievement will mean little. Accordingly, the Sustainable Development Goals include a focus on learning whereas the Millennium Development Goals focused largely on schooling. Another notable effort to measure and track learning on a global scale is the World Bank’s human capital index in which the levels of human capital of countries around the world are compared^2^. This effort aims to report measures of human capital that will encourage countries to invest in education. The human capital index includes learning outcomes from this database as one of its core components. The database in this Article will be updated regularly and made public to enable these large-scale efforts and to advance our understanding of the formation of human capital in developing economies.

## Methods

### Data reporting

No experiments were performed. No statistical methods were used to predetermine sample size of the harmonization of learning data or analyses done in this paper. The underlying microdata from the original learning assessments have detailed survey sampling procedures detailed in their corresponding technical reports.

### Test-score-linking methodology

We include 164 countries, two-thirds of which are developing countries, by linking international assessments to their regional counterparts. Regional assessments cover much of sub-Saharan Africa and Latin America but have often been excluded from international comparisons. We convert a regional test score to an international test score within subjects and schooling levels (primary and secondary) and within adjacent years. By including tests across the same testing round and at the disaggregated schooling and subject level, this method minimizes the likelihood that test differences are a function of time, proficiency, schooling level or data availability and maximizes the likelihood that they reflect test difficulty. We then apply this conversion to a country that participates in a regional test but not an international test to produce a comparable score (referred to as a HLO in the database).

The success of the linking approach hinges on three key assumptions. First, linked tests must capture the same underlying population. This assumption is satisfied by using sample-based assessments representative at the national level for cases in which a country participated in both a regional and an international assessment. This ensures that the underlying population tested is the same on average. Second, tests should measure similar proficiencies. To this end, we link within subjects (mathematics, reading and science) and schooling levels (primary and secondary) to ensure overlap. Third, the linking function should capture differences between tests rather than country-specific effects. This assumption is more likely to hold the larger the number of countries that participate in a given pair of tests being linked. To maximize the likelihood that this assumption holds, we construct the linking function over the entire interval. This step increases the sample size used to link tests, improving the likelihood that we capture test-specific rather than country-specific differences. In fixing the linking function, we assume that the relationship between tests stays constant across rounds. This assumption is reasonable since the mid-1990s, when assessments started to use a standardized approach and to link testing rounds with overlapping test items. A related advantage of a linking function over a fixed interval is that it guarantees that any changes in test scores over this interval are due to realized progress in learning rather than changing linking functions between tests. Of note, every update of the database increases the number of countries participating in a given pair of assessments. Thus, each update expands coverage and enhances the reliability of all estimates by enabling the construction of a more robust linking procedure.

We use multiple methods to link regional to international assessments. Our primary approach uses regression when multiple countries participate in the assessments being compared. When only one country participates, we use linear linking. Supplementary Information IIA describes both methods and the respective tests used. Both methods adjust test scores by a constant as well as by relative standard deviations across tests. These approaches build on a literature comparing scores across different tests^34,35^ as well as more recent work linking aggregate level scores across states in the USA^36^. In Supplementary Information IIB, we conduct a series of sensitivity tests, including conducting the conversion using country-fixed effects or random draws of countries and time periods. We further explore additional methods in Supplementary Information IIB, such as mean linking and ratio conversions, highlighting the trade-offs of each approach and examining robustness across them. We find a 0.99 or higher correlation coefficient for scores and relative ranks across all robustness tests (Supplementary Fig. 7). Limitations are described in Supplementary Information IIC. Detailed descriptions of all data sources are included Supplementary Information IID. Additional methodological parameters, such as the disaggregation of the data, are described in Supplementary Information IIE.

**Extended Data Fig. 4:**
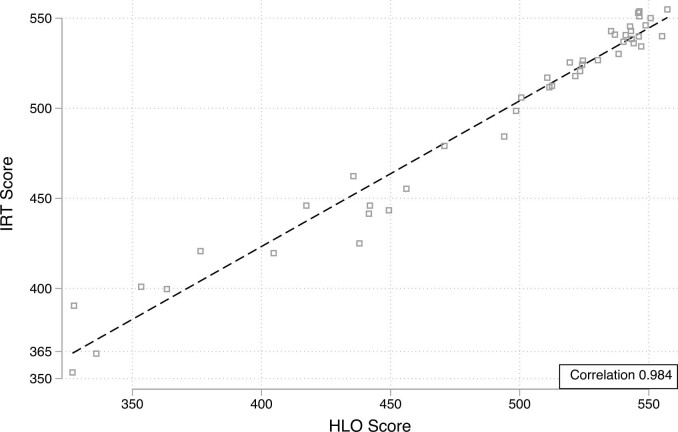
Comparison of HLOs to IRT. We compare average scores per country from our learning data for primary school from 2000 to 2010 to the LINCS project which uses IRT methods to link test score data. IRT methods are used to create scores for the underlying international and regional assessments used. The LINCS project produces scores on a globally comparable scale for primary school scores^40^.

We compare our data to a smaller database using item response theory (IRT)—in which tests share common test items—and find a 0.98 correlation coefficient (Extended Data Fig. 4). IRT—which is considered to be one of the most reliable methods to link tests in the psychometric literature—models the probability that a given pupil answers a given test item correctly as a function of pupil- and item-specific characteristics^34,37,38^. This methodology is used to construct the underlying tests that we use. To use it to compare learning across assessments, we need enough overlap in the underlying test items across assessments^39, 40^. However, such overlap does not exist for a large-enough set of tests and time periods that are needed to create a globally comparable panel dataset^40^. For example, TIMSS 1995 and Southern and Eastern Africa Consortium for Monitoring Educational Quality (SACMEQ) 2000 included overlapping mathematics items, but had only three test questions that would enable a comparison. When this overlap is small, standard maximum likelihood estimates will reflect both true variance and measurement error, overstating the variance in the test score distribution. The various challenges of estimating IRT parameters with limited item-specific overlap have previously been discussed in more detail^39^. Although IRT might not be a reliable approach when there is limited item-by-item overlap, we conduct comparisons in which the overlap is larger, with up to 17 common test items across tests. We compare our results to the Linking International Comparative Student Assessment (LINCS) project, which uses IRT methods and has an overlap in items for a subset of international studies focused on reading in primary schools^40^.

We compare the average scores for the same subject (reading), schooling level (primary) and time period (2000–2010) and find a correlation coefficient of 0.984 (Extended Data Fig. 4). This comparison indicates that even as we expand coverage to 164 countries in our database, we maintain high consistency with alternative measures for the subset of proficiencies, school levels and subjects for which there is overlap.

Of note, the assessments included in this database are conducted at school. Testing, and thus learning, data could be affected by enrolment patterns, and we advise users of the data to analyse learning outcomes alongside enrolment trends. For example, average test scores could be driven by lower-performing students entering the system rather than learning progress for those who were already in school. Although this is a potential concern when analysing average scores, there are several reasons why harmonized learning outcomes are still useful. First, primary enrolment rates are relatively high in all countries, reaching 90% on average. Second, learning measured with school-based tests is likely to yield a conservative upper bound of learning in a given country. As most countries at the bottom of the distribution of measured learning are also those with relatively low enrolments, it is unlikely that new school entrants would alter conclusions related to cross-country patterns—the lowest performing countries would probably be revealed to be performing even worse.

### Comparison of trends in schooling and learning

#### Expected years of schooling versus learning basic skills

Related to Extended Data Fig. 1. We define two key variables for analysis in this figure. First, we define our learning variable. Our measure of quality comprises the primary HLO scores, which measure the degree to which students acquire basic skills in primary school. Second, we define the schooling variable. The expected years of schooling measure is constructed by UNESCO (United Nations Educational, Scientific and Cultural Organization) and is a function of enrolment patterns and the number of years of schooling a given country formally provides. This measure is often interpreted by the international education community as a measure of a strong education system in which students attend many years of school. As the expected number of years of schooling is partially a function of enrolment, which we also use as a measure of schooling at times in this paper, these two measures are highly correlated. For countries with data available for both measures, we find an average correlation coefficient of 0.72 across them.

We find a high variance in learning conditional on years of schooling (Extended Data Figure 1). Ghana stands out as a country in which schooling is close to 12 years of schooling, yet the learning score is below the threshold of minimum proficiency of 300. Next, consider the examples of Zambia and South Africa. In Zambia, the average child is expected to have more than 9 years of schooling yet achieves a score of 301 on primary school learning. By contrast, in South Africa, with similar years of expected schooling, the average child scores 366. Given that both countries have more than 9 years of expected schooling, the primary-school learning scores are unlikely to be driven by selection. In addition, average primary enrolment rates over the 2000–2015 time period are high in both countries (98.2% in South Africa and 92.9% in Zambia). As learning outcomes are measured using tests taken in grades 4–6, primary school enrolment rates are relevant measures for schooling comparisons. Typically, large dropouts occur between schooling levels, such as the transition from primary to secondary school. However, enrolment up to the last grade within primary school is persistently high. For example, in South Africa, data from the World Bank show that 90% of students are retained until the end of primary school. This varies across contexts, but in many countries enrolment rates in primary school are relatively stable through the middle years of primary school when achievement tests are taken.

We further observe an exponential shape of the line of best fit, with a smaller correlation coefficient between schooling and learning for countries that provide 10 or fewer years of schooling on average than after this threshold (with a correlation coefficient of 0.35 relative to 0.73, respectively). The exponential curve is suggestive and should be viewed with a few caveats in mind. For example, it is conceivable that Brazil has higher learning scores than South Africa, not because the quality of education in Brazil is higher, but because of lower enrolments, which means that higher-ability students remain in the system at the time of the test. However, this is unlikely as Brazil has around 12 years of expected schooling and South Africa has around 9 years, meaning that most children progress through primary school and thus their primary-school learning scores largely reflect the quality of education rather than selection. The selection concern may be more pronounced at low levels of expected schooling. Even so, the flat part of the curve between 7 and 10 years of expected schooling is unlikely to reflect selection, given that learning is measured through tests in primary school, and primary school enrolment in countries with expected schooling of 7 or more years, is typically high. Moreover, the fact that learning levels vary substantially for a given point on the *x* axis reveals substantial heterogeneity in school quality even in systems in which the average student receives a similar number of years of education. These patterns suggest that schooling does not automatically translate to learning.

#### Regional learning and enrolment trends

The following section refers to methods used to compare trends in enrolment and learning over time. We restrict our comparison to countries with data points for at least two time periods for both enrolment and learning data in primary school to maximize comparability over the time period. The list of 72 countries included in the following analyses are: Argentina, Australia, Austria, Benin, Brazil, Bulgaria, Cameroon, Canada, Chile, Colombia, Costa Rica, Cyprus, Czech Republic, Côte d’Ivoire, Denmark, Dominican Republic, Ecuador, Finland, France, Gambia, Germany, Guatemala, Haiti, Honduras, Hong Kong, Special administrative regions of China (SAR China), Hungary, Iceland, Indonesia, Islamic Republic of Iran, Ireland, Italy, Japan, Jordan, Kenya, Korea (South), Kuwait, Lesotho, Liberia, Malawi, Malta, Mauritius, Mexico, Morocco, Mozambique, The Netherlands, New Zealand, Nicaragua, Niger, Norway, Panama, Paraguay, Peru, The Philippines, Poland, Portugal, Romania, Russian Federation, Senegal, South Africa, Spain, Eswatini (Swaziland), Sweden, Taiwan, Republic of China, Togo, Trinidad and Tobago, Tunisia, Turkey, Uganda, United States of America, Uruguay, Zambia, Zimbabwe. We average the HLO data into 5-year intervals to be analogous in structure to previously published primary enrolment data^29^. As our learning data extends to 2015 and enrolment data extends to 2010, we make a conservative assumption that enrolment rates persist through 2015 to enable inclusion of all learning data.

#### Regional learning and enrolment trends conditional on county-fixed effects

In Fig. 2, estimates are calculated controlling for country-fixed effects using multivariate regression by region. For each region and outcome *y* (primary enrolment and learning), we estimate the following specification: ${y}_{ct}^{r}=\,{\alpha }^{r}+{\beta }^{r}t+{{\boldsymbol{\delta }}}_{c}^{r}+{\varepsilon }_{ct}^{r}\,$ in which *t* represents the year and **δ** represents a vector of dummy variables for each country *c* in a given region *r*. On the basis of this regression, we recover regional time trends accounting for country-fixed effects.

#### Regional learning trends conditional on county-fixed effects and enrolment

In Extended Data Fig. 2, we further explicitly condition for enrolment effects and find nearly identical patterns to Fig. 2. For each region and outcome *y* we run the following specification: ${y}_{ct}^{r}={\alpha }^{r}+{\beta }^{r}t+{{\boldsymbol{\delta }}}_{c}^{r}+{\gamma }_{ct}^{r}+{\varepsilon }_{ct}^{r}$, in which *t* represents the year, **δ** represents a vector of dummy variables for each country *c* in a given region *r* and *γ* represents primary enrolment rates. We then recover regional time trends accounting for country-fixed effects and conditional on enrolment.

#### Enrolment and learning regression

We run the following regression: ${y}_{ct}=\,\alpha +\beta {\gamma }_{ct}+{{\boldsymbol{\delta }}}_{c}+{\varepsilon }_{ct}$ in which *c* represents a country, *t* represents a year, *γ* represents primary enrolment rates and **δ** represents a vector of dummy variables for each country *c*. The coefficient of interest is *β*, which captures the association between learning and enrolment. As noted in the ‘Schooling is not learning’ section, we find no statistically significant relationship between schooling and learning with a negative coefficient on enrolment rates of 0.247 with a *P* value of 0.673, reinforcing the patterns shown in Fig. 2 and Extended Data Fig. 2. We also find an *R*^2^ value of 0.96. We omitted four countries (Mozambique, Niger, Cameroon and Benin) that are outliers above the 95th percentile in enrolment changes, which can bias average cross-country trends. Another way to visualize the absence of an association between learning and enrolment is a scatter plot of learning and enrolment trends over time by country. This plot is provided in Supplementary Fig. 4.

#### Learning by country

Related to Extended Data Fig. 3. We illustrate regional patterns by focusing on a few specific examples from Latin and Central America, sub-Saharan Africa and the Middle East (Brazil, Colombia, Mexico, Uganda and Kuwait). Extended Data Figure 3 shows that learning in all of these countries has been consistently low and improved slowly over the past two decades, ranging from 360 to 453, which translates into many students not acquiring basic skills such as being able to read a simple story, even though all of these countries have achieved high enrolment rates above 99% in all years. Moreover, in each of the country examples, as primary enrolment rates are extremely high and flat, this reinforces that slow learning trends are not a function of enrolment. Figure 2 and Extended Data Fig. 2, in which country-fixed effects are controlled for, can be thought of as the generalizations of these patterns to the regional level.

### Development accounting

The contribution of human capital to cross-country income differences is analysed in studies of development accounting. We follow the literature^5,6,41^ and begin with a standard aggregate production function in its per-capita form:

$$y=Ah\,{\left(\frac{K}{Y}\right)}^{\frac{\alpha }{1-\alpha }}$$

in which *y* represents output per worker, *K* denotes capital, *h* denotes the level of human capital per capita and *A* captures the residual, typically interpreted as total factor productivity. Taking the logarithm on both sides decomposes cross-country income differences into three sources: capita–output ratio, average human capital and total factor productivity. Below, we only report the share of income differences that can be explained by variation in human capital, given that human capital is the focus of this paper. In Table 1 (top), we show decompositions that have used both human capital measures that incorporate education quality and education quantity^5,7,12^. In Table 1 (bottom), we include an additional decomposition, $\frac{\mathrm{ln}({h}_{90})-\,\mathrm{ln}({h}_{10})}{\mathrm{ln}({y}_{90})-\,\mathrm{ln}({y}_{10})}$, which has been used in studies that have used human capital measures that account for education quality^10,11,13^.

To measure human capital, we extend the standard Mincer specification that weights education by its micro-labour-market returns to include learning in addition to schooling:

$$h={{\rm{e}}}^{rS+wL}$$

in which *S* is the quantity of schooling, *L* is a measure of learning, and *r* and *w* are their respective returns. For years of schooling, we use previously published data^32^. For learning measures, we use the data presented in this paper. We assume rates of return on the basis of the microeconomic literature: we take the value *r* = 0.10 for the rate of return per school year, and *w =* 0.20 per standard deviation increase in learning, based on parameter values used in the literature^42,43^. The 0.20 value is based on US data. However, we can expect that returns to learning will be higher in developing countries in which the supply of skills is lower, as is the case with the returns to schooling literature^42^. A considerable number of studies have investigated these parameters. For the purpose of this paper, our intention is not to provide definitive results, but rather to motivate the use of the data in the development accounting literature. To this end, we take parameter values as given and conduct sensitivity analyses with values *w* = 0.15 and *w* = 0.25. We included 131 countries that have both schooling data^32^ and learning data.

We first compared our results to three previous studies^5,7,12^ in Table 1 (top). We find, in our estimates, that when the human capital measure only captures quantity (*w* = 0), human capital accounts for roughly 7–11% of the differences in output per worker. However, when we include measures of quality (*w* > 0), we find that this contribution increases to 14–21%. These results suggest that measuring human capital while taking into account quality substantially increases the role of human capital in explaining cross-country output per worker differences. These results are consistent with the literature showing that when quality is taken into account, the role of human capital in explaining cross-country differences in output per worker doubles^7^ relative to when only quantity is taken into account^5^.

In Table 1 (bottom), we show results focusing on $\frac{\mathrm{ln}({h}_{90})-\,\mathrm{ln}({h}_{10})}{\mathrm{ln}({y}_{90})-\,\mathrm{ln}({y}_{10})}$. The literature^10,11,13^ that has used this decomposition estimates that the contribution of human capital to cross-country income differences ranges from nearly 100% to close to 0%. We show that when we include our measure of quality, the share of human capital varies between 40% and 48%.

### Reporting summary

Further information on research design is available in the Nature Research Reporting Summary linked to this paper.

## Online content

Any methods, additional references, Nature Research reporting summaries, source data, extended data, supplementary information, acknowledgements, peer review information; details of author contributions and competing interests; and statements of data and code availability are available at 10.1038/s41586-021-03323-7.

### Supplementary information

Supplementary Information This file contains a Supplementary Discussion, Supplementary Methods, Supplementary References, Supplementary Tables 1-6 and Supplementary Figures 1-15.

Reporting Summary

## Figures and Tables

**Extended Data Table 1:**
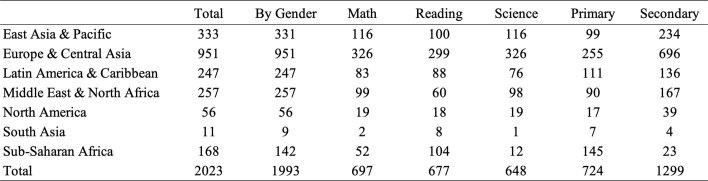
Country–year observations by disaggregation and region

Counts of country–year observations by region and disaggregation (schooling level and subject) are shown. The database includes 2,023 observations across all countries from 2000 to 2017.

**Extended Data Table 2:**
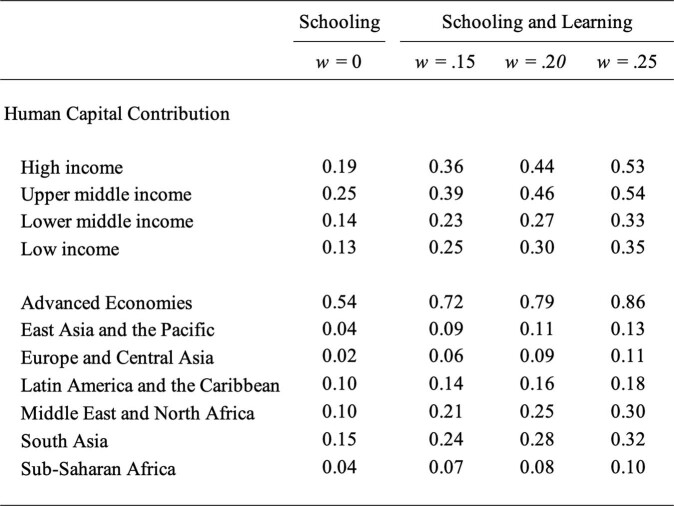
Human capital share by income status and region

The variable *y* is real output per worker on average from 2000 to 2010; *h* is a measure of human capital constructed on the basis of both schooling and learning estimates on average from 2000 onwards. We include the following decomposition of the role of human capital in explaining cross-country income differences on the basis of the literature^5–13^: var(log[*h*]/var(log[*y*]). Variable constructions and decompositions are described in detail in the Methods. We assume rates of return to the learning component of human capital, defined as *w*, on the basis of the microeconomic literature^42,43^. We conduct sensitivity analyses with values *w* = 0.15, *w* = 0.20 and *w* = 0.25. When *w* = 0, our accounting de facto only includes schooling; for any value *w* > 0, we include learning as well as schooling. We include 131 countries in this development accounting exercise. Schooling data are from a previously published study^32^. GDP data on real output per worker are from Penn world tables v.9.0^14^. Learning estimates are from our database.

## Data Availability

The data are available for public use and updated regularly on the World Bank website: https://datacatalog.worldbank.org/dataset/harmonized-learning-outcomes-hlo-database. The database is expected to be updated every 2–3 years as new learning data become available. The database will be updated at the same location on the World Bank website using the methodology and approach in this paper, with accompanying technical notes on additional countries and data sources added. This study used a combination of data sources, including data that are available from online repositories and required straightforward registration and usage agreement. We also collected data from over 48 countries directly in collaboration with the World Bank and USAID.

## Code availability

The code used to compile and analyse data was written in Stata v.15.1. The code to replicate analysis and tables is available from GitHub (https://github.com/measuringhumancapital).
