# Supplementary material for: Measuring human capital using global learning data
Source: Nature. 2021 Mar 10;592(7854):403–8. doi: 10.1038/s41586-021-03323-7 (PMC8046667; doi:10.1038/s41586-021-03323-7)
Supplement: Supplementary file 1 — This file contains a Supplementary Discussion, Supplementary Methods, Supplementary References, Supplementary Tables 1-6 and Supplementary Figures 1-15. [file 41586_2021_3323_MOESM1_ESM.pdf]

nature portfolio

https://doi.org/10.1038/s41586-021-03323-7

Supplementary information

Measuring human capital using global learning data

In the format provided by the authors and unedited

Measuring Human Capital using Global Learning Data - Supplement

Noam Angrist, Simeon Djankov, Pinelopi K. Goldberg, and Harry A. Patrinos

Table of Contents

I. Supplemental Discussion ... 2

A. Descriptive Statistics Supplement ... 2

II. Supplemental Methods ... 7

A. The Linking Procedure ... 7

B. Sensitivity Tests ... 9

C. Potential Limitations ... 13

D. Supplemental Data Description ... 14

1. International Standardized Achievement Tests (ISATs) ... 14

2. Regional Standardized Achievement Tests (RSATs) ... 15

3. The Early Grade Reading Assessment (EGRA) ... 17

4. Summary of Assessments Included in the Database ... 18

E. Additional Methodological Parameters ... 19

Supplemental References ... 21

Supplemental Tables ... 21

Supplemental Figures ... 22

I. Supplemental Discussion

A. Descriptive Statistics Supplement

Supplement Table 1 presents country-subject-level observations by year. The data are spread over time, slightly weighted towards recent years since countries are increasingly participating in assessments. A related feature of the data is a large influx of data in particular testing years. This is more prevalent for developing regions which participate in more sporadic assessment. While our database presents the largest coverage of learning data across countries and over time, data availability remains sporadic.

Supplement Table 1 | Country-subject-level observations by year

Year	Total	Female	Male	Math	Reading	Science	Primary	Secondary
2000	155	155	155	56	56	43	26	129
2001	33	33	33	0	33	0	33	0
2002	2	2	2	0	2	0	2	0
2003	221	221	221	90	41	90	44	177
2004	1	1	1	0	1	0	1	0
2006	262	262	262	73	123	66	92	170
2007	193	193	193	96	15	82	97	96
2008	3	3	3	0	3	0	3	0
2009	226	225	225	74	79	73	7	219
2010	6	5	5	0	6	0	6	0
2011	240	240	240	92	56	92	152	88
2012	202	201	201	64	74	64	10	192
2013	78	54	54	27	36	15	78	0
2014	19	18	18	0	19	0	19	0
2015	323	323	323	125	74	123	96	227
2016	55	54	54	0	55	0	55	0
2017	4	3	3	0	4	0	4	0
Total	2023	1993	1993	697	677	648	725	1298

Notes: This table presents country-subject-level observations by year.

Supplement Figure 1 summarizes learning for a subset of countries in a boxplot showing medians and interquartile ranges using raw data from 2007-2017. We observe a few interesting case studies. Russia (519) performs similarly to the United States (524). Chile (450) outperforms Eastern European countries such as Georgia (445). Saudi Arabia (393) places near the bottom outperforming only Egypt (372), Morocco (350) and Rwanda (308). The gap between Morocco (350) and Singapore (570) is substantial. Singapore and Finland (552) have low variation due to a potential plateau on the upper end of performance. Rwanda has low variation due to limited data. Russia has high variation due to improving learning, whereas South Africa has high variation due to declining learning.

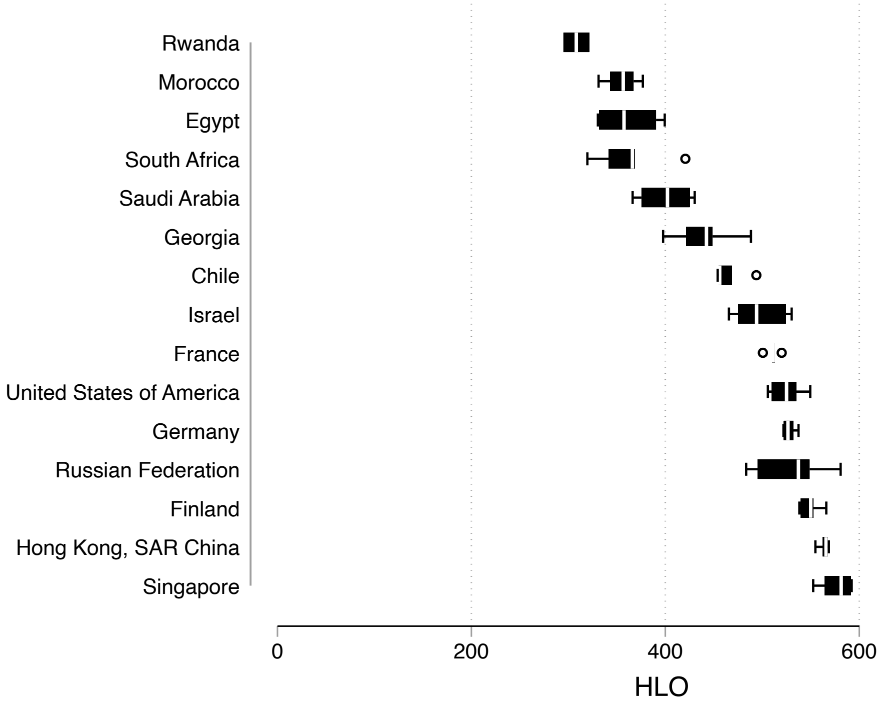

Supplement Figure 1 | Learning (2007-2017) – selected countries

Notes: Learning estimates are average harmonized learning outcomes per year (across subjects and schooling levels) from 2007 to 2017. The boxplot plots the distribution of average learning over the time period, showing median HLO scores and the interquartile ranges.

Supplement Figure 2 plots average learning levels from 2000-2017 for each country side-by-side with the log of their GDP per capita. This graph illuminates cases where countries have managed to improve learning despite a lack of resources, as well as cases where countries have resources to invest in to date unrealized learning potential. Former or current centrally planned economies display better learning outcomes than their income would suggest (and accordingly are above the line of best fit), such as Singapore, Poland, Bulgaria, Cuba and Vietnam. Countries in the Middle East and Africa reach lower learning levels than predicted by income (and accordingly are below the line of best fit), such as Qatar, Kuwait, United Arab Emirates, South Africa, Nigeria and Ghana. We also highlight large developing countries: India, China, Mexico, and Brazil. China outperforms its counterparts, Mexico, India and Brazil perform slightly below where their income would predict, and South Africa trails far behind.

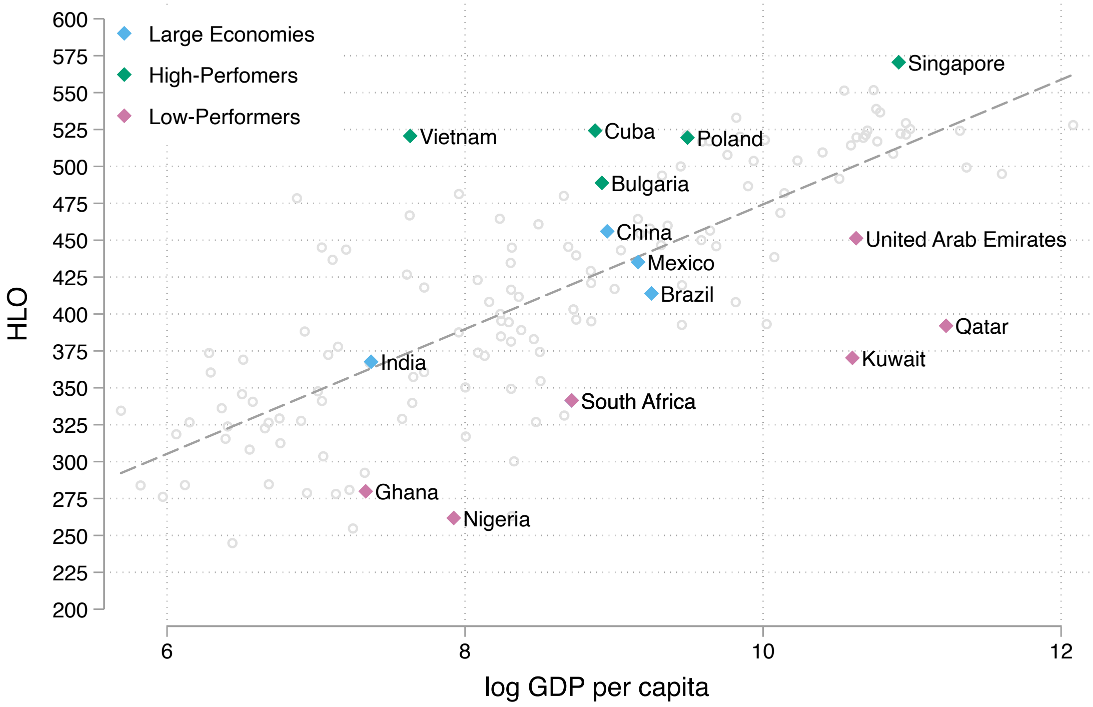

Supplement Figure 2 | Average Learning (2000-2017) versus 2015 GDP per capita

Notes: Average learning is calculated across subjects and schooling levels over the given time period from 2000 to 2017. GDP per capita estimates are from World Bank national accounts data; learning outcomes are from our database.

Next, we explore gender gaps. In Supplement Figure 3 we show average learning levels per region by gender from 2000 to 2010. We find gender gaps in learning are positive on average with girls outperforming boys across nearly all regions. This points in the opposite direction of the gender gap for years of schooling which is negative on average.

This might suggest that as women increasingly join the labor market worldwide, girls who have attained schooling might realize large returns if they can obtain skilled work and might partially explain why in cross-country Mincerian returns estimates women have higher returns to schooling.[42] Of note, the flip in the gender gap might be due to selection. In regions where enrollment is low, only high achievers might be taking assessments. This explanation is consistent with trends observed for the Middle East and North Africa. However, in Sub-Saharan Africa, where the enrollment gap is second lowest (with 5.3 percentage point less enrollment for females), the learning gender gap is low and negative (with negative .9 percentage point less learning among females), as is the enrollment gap, indicating selection is unlikely the only driver. We present the contrast in gender gaps in schooling versus learning not as definitive, but rather to motivate further in-depth exploration, which we hope this database can enable.

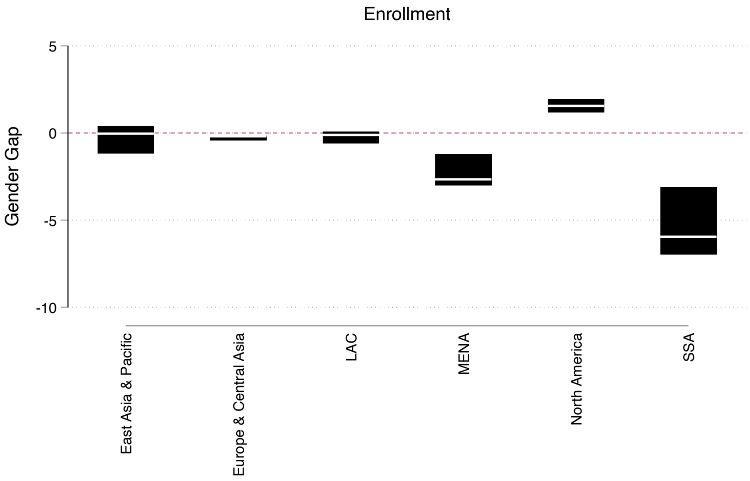

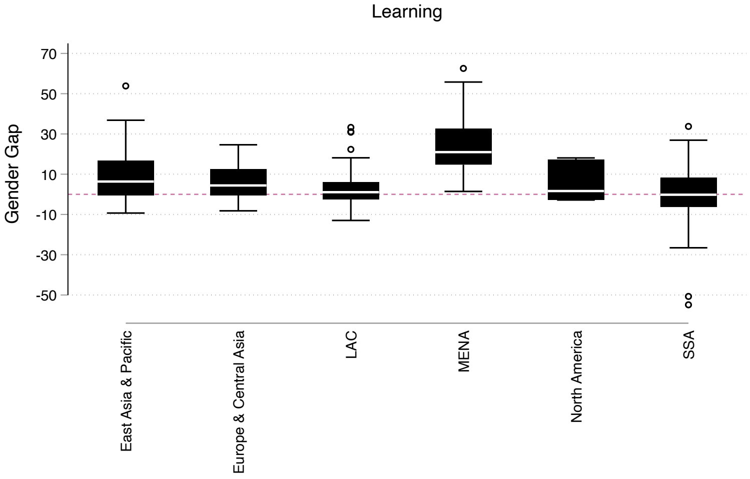

Supplement Figure 3 | Gender gap – enrollment versus learning (2000-2010), by region

Notes: The gender gap takes the average difference of female and male enrollment or learning per region in a given time period. The boxplot shows the distribution of gender gaps over all time periods per region, plotting the median and interquartile range. A positive gender gap indicates females do better and vice-versa. LAC refers to Latin American and the Caribbean; MENA refers to the Middle East and North Africa; and SSA refers to Sub-Saharan Africa. Primary enrollment rates are from Lee and Lee (2016).[29] Learning estimates are taken from our database.

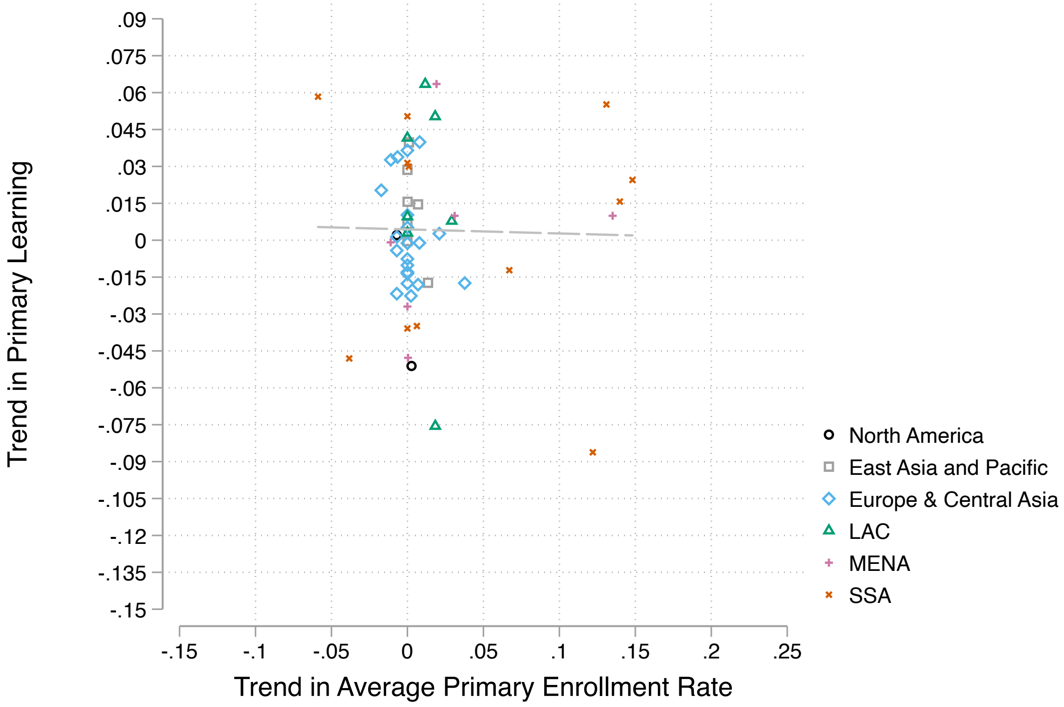

Supplement Figure 4 | Trends in primary enrollment versus primary learning (2000-2015), by region

Notes: Trends are calculated using the annualized difference between consecutive time periods, and then averaged over the entire interval from 2000 to 2015. We express learning and enrollment in terms of standard deviations for comparable units. LAC refers to Latin American and the Caribbean; MENA refers to the Middle East and North Africa; and SSA refers to Sub-Saharan Africa. We omit four countries (Mozambique, Niger, Cameroon and Benin) who are outliers above the 95th percentile in enrollment changes which can bias average cross-country trends. Primary enrollment rates are from Lee and Lee (2016).[29] Learning estimates are taken from our database.

We extend the analysis in the main text and further explore the relationship between schooling and learning. In Supplement Figure 4 we show a scatterplot of trends in progress in average primary enrollment and in learning in primary school over the last decade. We measure schooling using adjusted enrollment ratios (Lee and Lee 2016).[29] We compare this measure of schooling to our measure of learning in primary school for the years 2000-2015. We use data for this period since it has the most overlap of schooling and learning measures. We restrict our comparison to countries with at least two data points for both enrollment and learning data in primary school to maximize comparability over the time period.

Trends are calculated using the annualized difference between consecutive time periods, and then averaged over the entire interval from 2000 to 2015. We express learning and enrollment in terms of standard deviations for comparable units.

We find a flat average relationship, revealing that schooling and learning do not necessarily improve together. The coefficient on the line of best fit between annual gains in enrollment and learning is -.017 with a p-value of .879, revealing no significant relationship. This insight suggests that policy could focus on improving *both* schooling and learning, rather than only focusing on only one of the two, in the hopes that the other will improve in turn.

II. Supplemental Methods

A. The Linking Procedure

The central intuition behind the construction of globally comparable learning outcomes is the production of a linking function between international and regional assessments. This function can be produced for countries that participate in a given pair of assessments and captures the difference in difficulty between the two assessments. This linking function can then be used to place scores for countries that only participate in regional assessments on the international scale. This enables construction of globally comparable learning outcomes.

We use multiple methods to produce globally comparable scores. Our primary approach uses regression when multiple countries participate in assessments being compared. When only one country participates, we use linear linking. Both methods adjust test scores by a constant as well as relative standard deviations across tests. These approaches build on a literature comparing scores across different tests[34,35] as well as a more recent work linking aggregate level scores across states in the United States.[36]

The conversion can be implemented by regressing mean scores from countries that partake in a regional and international assessment to derive α and β and produce a linking function between assessments:

$$\mu_{Yi} = \alpha + \beta\mu_{Xi} + \varepsilon_i$$

where μ denotes the mean scores, X is a regional assessment, Y is an international assessment and i denotes countries that have scores on both assessments. We can then convert scores from countries that only participate in regional assessment X onto an international scale Y using α and β.

The success of this approach hinges on three key assumptions. First, linked tests must capture the same underlying population. This assumption is satisfied by using sample-based assessments representative at the national level where a country participated in both a regional and international assessment. This ensures that the underlying population tested is the same on average and we capture differences between tests.

Second, tests should measure similar proficiencies. To this end, we link within subjects (math, reading and science) and schooling levels (primary and secondary) to ensure overlap.

Third, the linking function should capture differences between tests rather than country-specific effects. This assumption is most likely to hold the larger the number of countries which participate in a given pair of tests being linked. To ensure this last assumption holds, we use the same linking parameters over the entire interval. Supplement Table 6 shows how tests that are linked over the entire interval accordingly. This increases the sample size used to link tests, increasing the likelihood that we capture test-specific rather than country-specific differences. In fixing the linking function over time, we assume that the relationship between tests stays constant across rounds. This assumption is reasonable since the mid-1990s when assessments started to use a standardized approach and to link testing rounds with overlapping test items. A related advantage

of fixing the linking function is that it guarantees that any changes in test scores over this interval are due to realized progress in learning rather than changing relationship between tests. Of note, every update of the database increases the number of countries participating in a given pair of assessments. Thus, each update both expands coverage as well as enhances the reliability of all estimates by enabling construction of a more robust linking procedure.

Below we capture a level of precision needed to satisfy the above assumptions. We produce a linking function within subjects and schooling levels (primary and secondary) from test X to test Y:

$$\mu_{Yisl} = \alpha + \beta\mu_{Xisl} + \varepsilon_{isl} \tag{1}$$

where i is a country in the set countries that participate in both tests X and Y in a given subject s, and schooling level l. Scores from test X and Y are further matched by testing round. We consider tests to be in the same round if they are five years apart and optimize to have the rounds as tight as possible. In all cases the time window is within four years. This minimizes the likelihood that test differences are a function of time, proficiency, schooling level, or data availability and are an accurate reflection of test difficulty.

We present a simplified and illustrative example. In 2006 Colombia and El Salvador participated in the regional test in Latin America and the Caribbean called LLECE as well as an international test, TIMSS. Thus, they have primary science scores on both assessments which are representative at the national level. In 2013, Chile and Honduras participated in both assessments and have primary science scores on both assessments which are representative at the national level. A regression for this set of countries of LLECE on TIMSS at primary level and on math scores yields an estimate β of .816 and a constant adjustment α of 15.824. We can then use this estimated relationship to convert scores from countries which only took part in regional assessments to an international scale. For example, Argentina has a score of 501.32 in primary science in 2013 on LLECE and would thus have an equivalent international score of 425.

We can also use an alternative approach called linear linking when only one country participates in pairwise assessments. This approach uses information on within-country standard deviations and mean scores to estimate α and β as follows:

$$Y = \alpha + \beta X \tag{2}$$

where $\alpha = \mu_Y - \beta\mu_X, \beta = \frac{\sigma_Y}{\sigma_X}$, and σ denotes within-country standard deviations on test X and Y. Both methods adjust test scores by a constant as well as relative standard deviations across tests. Supplement Table 6 shows the number of linking countries for each test being linked. The relevant method is used accordingly.

By producing a linking function and placing regional scores on an international scale, we are able to compare learning outcomes on a global scale. On this scale, 625 represents advanced attainment and 300 represents minimum attainment. This interpretation is derived by taking established benchmarks already used on international and regional assessments. For the high-performance benchmark on the upper end of the distribution, we use the TIMSS benchmark of 625. For the low-

performance benchmark on the lower end of the distribution, we use 300, which is the equivalent on the HLO scale of the minimum benchmarks on regional assessments such as LLECE and PASEC. This approach enables us to capture performance across the distribution and accounts for floor and ceiling effects that would be introduced by taking either international or regional benchmarks on both ends of the distribution. Supplement Table 6 includes descriptions of each assessment to enable derivation of linking functions.

B. Sensitivity Tests

We conduct a series of sensitivity tests. First, we examine the degree to which linking functions are stable across countries using two approaches. For tests where we have multiple participating countries and for which we use the regression method we can also produce linking functions using country-fixed effects. This modifies linking equation (1) above as follows:

$$\mu_{Yisl} = \alpha + \beta\mu_{Xisl} + \delta_c + \varepsilon_{isl} \quad (3)$$

where δ_c is a strata dummy for country-fixed effects. Supplement Table 2 compares scores with and without country-fixed effects. We observe differences in scores ranging from 3 to 15 points. The differences in scores are relatively small. One method to quantify these differences is to put them in terms of standard errors. In Supplement Figure 6 we observe that standard errors are on average 3.6 points with a range of up to 18 points. Thus, these differences fall broadly within the range of error. Moreover, we find a perfect correlation among scores within test and subject.

Supplement Table 2 | Scores using regression with and without country-fixed effects

	(1)<br>EGRA reading	(2)<br>LLECE math	(3)<br>LLECE reading	(4)<br>LLECE science	(5)<br>PISA math	(6)<br>PISA science
HLO	329.8	383.3	454.1	423.2	489.8	496.0
HLO - Country Fixed Effects	326.5	368.4	462.7	414.5	476.8	488.1
Correlation	1.000	1.000	1.000	1.000	1.000	1.000

Notes: HLO references Harmonized Learning Outcomes produced with a linking function without country-fixed effects per equation (1) as follows: $\mu_{Yisl} = \alpha + \beta\mu_{Xisl} + \varepsilon_{isl}$. HLO- Country Fixed Effects refers to HLO scores produced from with a linking function derived using a regression which includes country-fixed effects per equation (3) as follows: $\mu_{Yisl} = \alpha + \beta\mu_{Xisl} + \delta_c + \varepsilon_{isl}$. We only compute scores using the regression method for LLECE, EGRA and PISA since SACMEQ and PASEC only have a single country used to make score comparisons and use linear linking.

We further test the robustness of linking by conducting a random draw of half of all available countries and time periods per test-subject-level to produce the linking function using linear linking for consistency of method. Supplement Figure 5 shows a scatter plot of scores with all countries and time periods relative to linking functions using a random sample. Supplement Table 3 quantifies these differences.

We find average point differences of less than 1 point for PISA, followed by 2 points for LLECE, 8.5 points for EGRA, 19 points for SACMEQ and 25 points for PASEC. This variation is consistent with the assumption suggesting smaller differences where there is more country overlap

and data availability. EGRA and LLECE converge similarly to PISA with the difference in scores falling within standard error margins of 1 to 10 points. PASEC and SACMEQ score differences vary more widely, necessitating caution when interpreting precise scores. Overall, we find consistently high correlations above .95 indicating while scores are not identical, they change in consistent directions. This indicates relative rankings and country groupings are preserved.

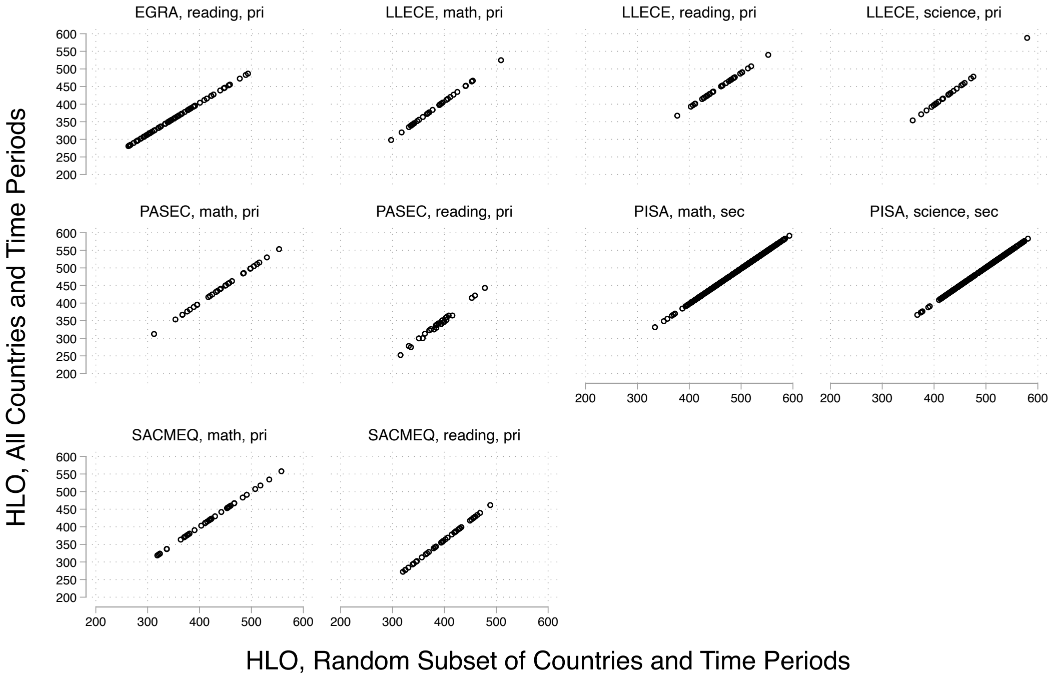

Supplement Figure 5 | Learning scores with all countries and time periods vs. random subset

Notes: We compare Harmonized learning Outcomes (HLO) using all county and time periods over the fixed linking period with HLO scores computed using a random subset of half of available countries for each test-subject-level as per equation (2).

Supplement Table 3 | Learning scores with all country and time periods vs. random subset

	(1)	(2)	(3)	(5)	(6)
	EGRA	LLECE	PASEC	PISA	SACMEQ
HLO	359.3	420.4	390.3	497.7	390.6
HLO - Random Set of Countries and Time Intervals	350.8	422.2	414.8	498.2	409.5
Correlation	1.000	0.990	0.953	1.000	0.955

Notes: We compare Harmonized learning Outcomes (HLO) using all county and time periods over the fixed linking function period with HLO scores computed using a random subset of half of available countries for each test-subject-level as per equation (2).

Next, we explicitly account for linking errors by including measures of uncertainty to quantify the degree of confidence around our estimates by test. We capture two sources of uncertainty: scores on the original test and uncertainty in the estimation of linking parameters across tests. We calculate the variance by bootstrapping. We consider each average country score on a given subject, test, and schooling level as a random variable with a mean – the score itself – and a standard deviation which captures the sampling variation across students. This distribution of scores is asymptotically normal by virtue of the central limit theorem. We take 1,000 draws from the distribution of subject-level average test scores for each testing regime. We do this as a computational shortcut, rather than bootstrapping subsamples of students from each test. We derive the linking function using linear linking for uniformity across all tests and scores from each bootstrapped sample. We find small uncertainty intervals overall, as shown in Supplement Figure 6 with an average of 3.6 points and ranging from 1 to 18 points. Consistent with sensitivity tests, we find larger uncertainty for our estimates relative to original scores when testing regimes have fewer countries participating in a given pair of tests. Supplement Figure 6 decomposes standard errors due to within-test sampling variation as well as variance in the linking function. This figure shows that for tests where there is no need to produce a linking function, or many pair-wise countries which we can use to produce this linking, the final standard errors remain similar to standard errors from the original test (such as PISA). For tests with fewer pair-wise countries, the linking has more uncertainty, such as PASEC, where the average standard error increases from 5.3 on the original test to 10 for the HLO. By quantifying the degree of uncertainty, we can more reliably bound our estimates.

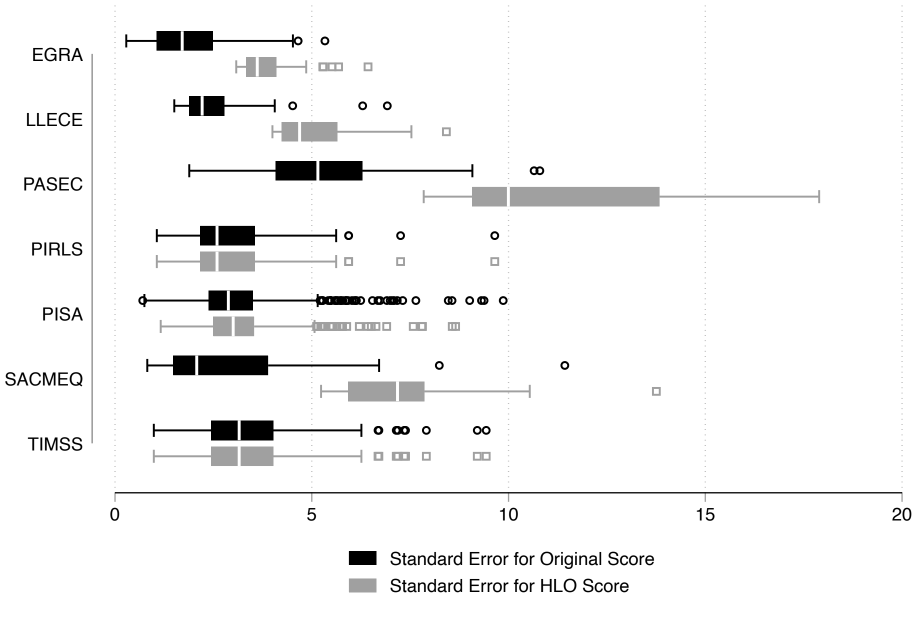

Supplement Figure 6 | Standard errors by test

Notes: We decompose standard errors on the overall HLO score versus the original test. We capture two sources of uncertainty: scores on the original test and uncertainty in the estimation of linking parameters across tests. We calculate the variance by bootstrapping. We consider each average country score on a given subject, test, and schooling level as a random variable with a mean – the score itself – and a standard deviation which captures the sampling variation across students. This distribution of scores is asymptotically normal by virtue of the central limit theorem. We take 1,000 draws from the distribution of subject-level average test scores for each testing regime. We do this as a computational shortcut, rather than bootstrapping subsamples of students from each test. We derive the linking function and scores from each bootstrapped sample.

We compare our primary linking approach using regression and linear linking with two alternative approaches and compare robustness across them in Supplement Figure 7. First, we use simple mean linking which introduces a constant adjustment between tests matched with rounds, and which we average across testing rounds. This approach assumes constant standard deviations across tests. Second, we use a ratio between test means and also take an average across rounds. This approach assumes a constant scalar adjustment λ between means and standard deviations across tests. The ratio approach is salient and intuitive for policymakers. However, a potential challenge in applying ratios is that they are in principle sensitive to the scale of the test. For example, given score scales have no absolute zero, in theory we can add 300 points to the mean of each test and preserve the interval properties of the scale, but will alter the conversion ratios (i.e., exchange rates). We address this potential issue by having strict inclusion criteria for the underlying tests: they have a uniform scale with a mean of 500 and standard deviation of 100. "Exchange rates" are derived using the same scale and applied on the same scale. Thus, while in theory changing score scales might bias results, by design this is not the case. This increases the likelihood we capture differences in test difficulty rather than arbitrary scaling variation.

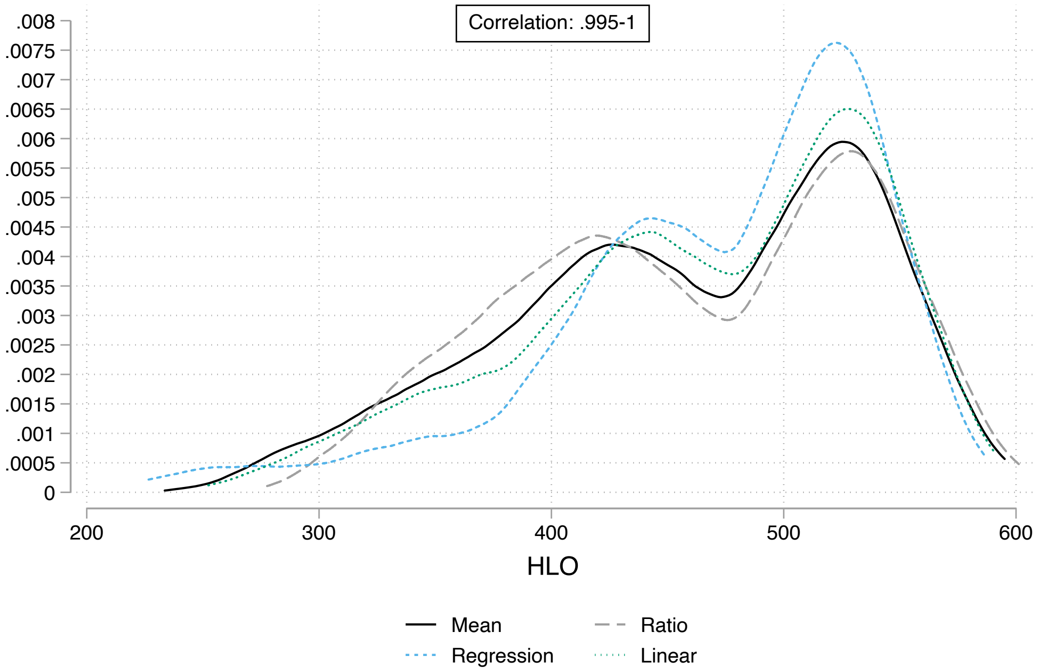

Supplement Figure 7 | Comparison of scores across linking methods

Notes: We compute Harmonized Learning Outcomes scores using multiple methods including regression, linear, mean and ratio linking. This figure compares scores using a density plot of scores using each method and the correlation coefficient across all methods. We compare our primary linking approach using regression and linear linking with two alternative approaches and compare robustness across them. A third approach is simple mean linking which introduces a constant adjustment between tests matched with rounds, and which we average across testing rounds. This approach assumes constant standard deviations across tests. A fourth approach we use is a ratio between test means and also take an average across rounds. This approach assumes a constant scalar adjustment λ between means and standard deviations across tests. The linear, ratio, and mean methods apply to all tests. For the regression method, this applies to all tests except SACMEQ and PASEC which only have a single country used to make score comparisons.

Supplement Figure 7 shows how scores compare across methods. Overall, we find a correlation coefficient of .995 across all methods and above, indicating high levels of robustness. Supplement Figure 8 below breaks down score distributions by both test and method. This reveals similar patterns, with testing regimes with more overlapping countries showing more consistent scores across method. Taken together, these results reveal overall robustness. A caveat is that scores from regional assessments from PASEC and SACMEQ in particular should be interpreted carefully and focus less on precise scores and more on relative ranks and country groupings.

Over time, as more countries participate in more assessments, we anticipate the linking functions used to produce harmonized scores will become increasingly robust. The approach outlined here produces a first set of global comparisons, demonstrates aggregate reliability, quantifies uncertainty to bound estimates, and provides a foundation for continuingly generating more robust data and comparisons as more countries partake in regional and international assessments.

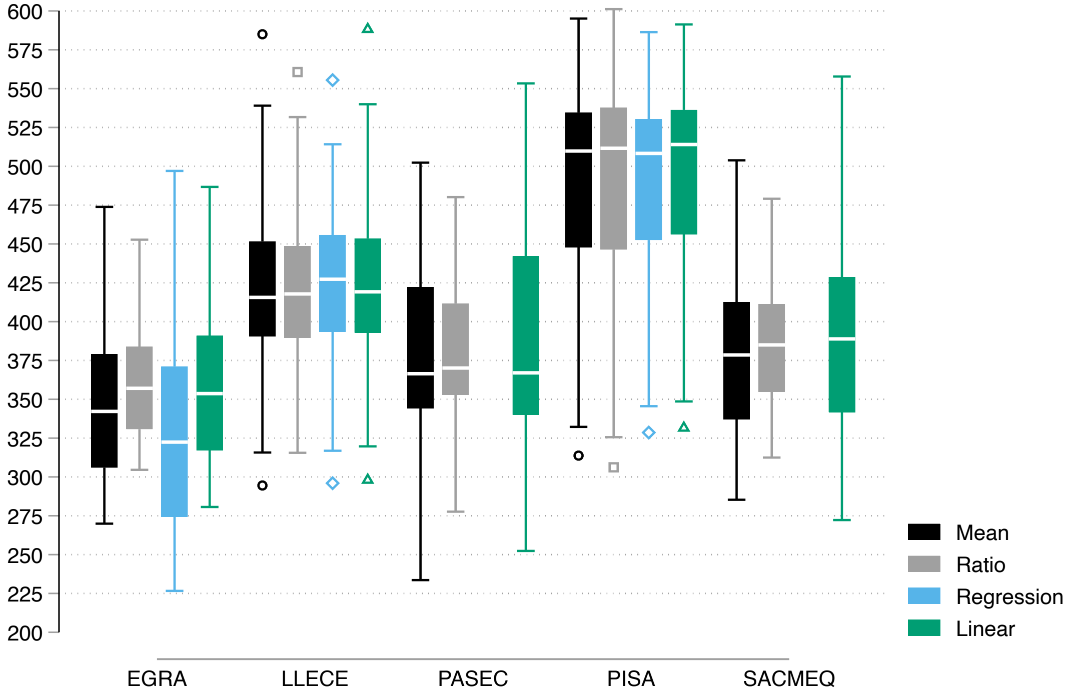

Supplement Figure 8 | Comparison of scores across linking methods by test

Notes: We compute Harmonized Learning Outcomes scores using multiple methods including regression, linear, mean and ratio linking functions. This figure compares scores using a density plot of scores using each method by source test. We compare our primary linking approach using regression and linear linking with two alternative approaches and compare robustness across them. A third approach is simple mean linking which introduces a constant adjustment between tests matched with rounds, and which we average across testing rounds. This approach assumes constant standard deviations across tests. A fourth approach we use is a ratio between test means and also take an average across rounds. This approach assumes a constant scalar adjustment λ between means and standard deviations across tests. The linear, ratio, and mean methods apply to all tests. For the regression method, this applies to all tests except SACMEQ and PASEC which only have a single country used to make score comparisons.

C. Potential Limitations

A potential limitation is the representativeness of the data of the total stock of cognitive skills in a given country. While the tests used for linking are nationally representative, they are conducted at the school. To this end, learning data might be affected by enrollment patterns, and we advise users

of the data to analyze learning outcomes alongside enrollment trends. For example, as marginal students enter the schooling system, average test scores might be driven by selection rather than true learning progress. While this is a potential concern, it is mitigated for a few reasons. First, primary enrollment rates are relatively high, reaching 90 percent on average, and above 75 percent even in the furthest behind regions, such as Sub-Saharan Africa. Second, the direction of the bias is likely to yield a conservative upper bound of learning in a given country. If all students enrolled, the average test score would be even lower, since the marginal students would pull the average down. Since most countries at the bottom of the distribution of learning are also those with relatively lower enrollments, it is unlikely this will alter substantive conclusions – the lowest preforming countries will be revealed to be even lower performing. In addition, data at the primary level should be largely unaffected, since at this level students are being taught basic skills, such as reading "the name of the dog is Puppy." Thus, even if marginal students enter the system, these students should still be expected to attain basic skills by the time they are tested in later primary school grades. Of note, in future work, we aim to include household-based learning data to sign and quantify the degree of selection present in school-based testing. However, current household-based data is limited and not yet comparable across a significant number of countries.

A second limitation regards data availability. While this is the largest learning outcomes database to date, data are still sparse for some countries. This introduces bias if data availability is correlated with education quality or progress. For example, if countries that perform worse have data only in later years (because they were later to introduce assessments), their average score will be likely biased upwards, as the test scores will reflect more recent testing, not stronger performance. Since we provide year-by-year scores this can be accounted for.

Relatedly, when averaging data across subjects, levels and over time, there is a possibility that averages reflect the availability of data rather than learning gains. For example, let's examine a case where a country has a score of 500 in 2000 in math and jumps to 550 in 2005. If this country added reading in 2005 and scored 450, the average score across subjects in 2005 would be 500, reflecting no learning progress since average scores would be 500 in both years. However, an apples-to-apples comparison in math shows learning grains from 500 to 550. To address this issue, we construct disaggregated measures by subject and schooling levels as well as aggregated ones. This enables analyses at each level considering the trade-offs.

A point of emphasis is that while learning measures human capital better than prior proxies, such as enrollment, learning does not capture the concept of human capital in its totality. Moreover, assessments do not capture only cognitive skills. For example, recent evidence suggests test scores pick up as differential effort as well as cognitive ability.[45] We use learning outcomes in this paper with these caveats in mind.

D. Supplemental Data Description

1. International Standardized Achievement Tests (ISATs)

In the mid-1990s, there was an emergence of standardized, psychometrically robust and relatively consistent ISATs. Below we describe the major ISATs we use in this database. All ISATs are

designed to be nationally representative. In cases where there are exceptions, the testing agencies note these with an asterisk, and we preserve this information in the database.

TIMSS. The Trends in International Mathematics and Science Study (TIMSS) is conducted by the IEA. Five TIMSS rounds have been held to date in Math and Science subjects covering grades 4 and 8. The first, conducted in 1995, covered 45 national educational systems and three groups of students. IEA assessments define populations relative to specific grades, while PISA assessments focus on the age of pupils. In IEA studies, three different groups of pupils were generally assessed: pupils from grade 4, grade 8 and from the last grade of secondary education. In 1995, two adjacent grades were tested in both primary (3-4) and secondary schools (7-8). To obtain comparable trends, we restricted the sample to grades 4 and 8. Some Canadian provinces and states in the United States of America have occasionally taken part in the IEA surveys.

The second round covered 38 educational systems in 1999, examining pupils from secondary education (grade 8). The third round covered 50 educational systems in 2003, focusing on both primary and secondary education (grades 4 and 8). In 2007, the fourth survey covered grades 4 and 8 and more than 66 educational systems. In 2011, the survey covered 77 educational systems across grades 4 and 8. The last round was performed in 2015 and covered 63 countries/areas. The precise content of the questionnaires varies but remains systematic across countries.

PIRLS. The Progress in International Reading Literacy Study (PIRLS) survey is also conducted by the IEA. The PIRLS tests pupils in primary schools in grade 4 in reading proficiency. Four rounds of PIRLS have been held to date in 2001, 2006, 2011 and 2016.

In 2006, PIRLS included 41 countries/areas, two of which were African countries (Morocco and South Africa), 4 lower middle-income countries (Georgia, Indonesia, Moldova, Morocco) and 8 upper middle-income countries (Bulgaria, Islamic Republic of Iran, Lithuania, Macedonia, Federal Yugoslavian Republic, Romania, Russian Federation, South Africa). The 2011 round of PIRLS was carried out alongside TIMSS and included 60 countries/areas. The newest round of PIRLS in 2016 includes 50 countries.

PISA. The Organization for Economic Co-operation and Development (OECD) launched the Programme for International Student Assessment (PISA) in 1997 to provide comparable data on student performance. Since 2000, PISA has assessed the skills of 15-year-old pupils every three years. PISA concentrates on three subjects: mathematics, science and literacy. The framework for evaluation remains the same across time to ensure comparability. In 2009, 75 countries/areas participated; in 2012, 65 countries/areas participated and in 2015, 72 countries/areas participated. An important distinction between PISA and IEA surveys is that PISA assesses 15-year-old pupils, regardless of grade level, while IEA assessments assess grade 4 and 8.

2. Regional Standardized Achievement Tests (RSATs)

In addition to the above international assessments, a series of regional assessments have been conducted in Africa and Latin America and the Caribbean. All RSATs are designed to be nationally representative. In cases where there are exceptions, the testing agencies note these with an asterisk, and we preserve this information in the database.

*SACMEQ***.** The Southern and Eastern Africa Consortium for Monitoring Educational Quality (SACMEQ). SACMEQ is a psychometrically designed, standardized test which generally assesses math, reading and English in grade 6 pupils. The first SACMEQ round took place between 1995 and 1999. SACMEQ I covered seven different countries and assessed performance only in reading. The participating countries were Kenya, Malawi, Mauritius, Namibia, United Republic of Tanzania (Zanzibar), Zambia and Zimbabwe. The studies shared common features (instruments, target populations, sampling and analytical procedures). SACMEQ II surveyed pupils from 2000-2004 in 14 countries: Botswana, Kenya, Lesotho, Mauritius, Malawi, Mozambique, Namibia, Seychelles, South Africa, Swaziland, Tanzania (Mainland), Tanzania (Zanzibar), Uganda, and Zambia. Notably, SACMEQ II also collected information on pupils' socioeconomic status as well as educational inputs, the educational environment and issues relating to equitable allocation of human and material resources. SACMEQ II also included overlapping items with a series of other surveys for international comparison, namely the *Indicators of the Quality of Education* (Zimbabwe) study, TIMSS and the 1985-94 IEA *Reading Literacy Study*. The third SACMEQ round (SACMEQ III) spans 2006-2011 and covers the same countries as SACMEQ II plus Zimbabwe. SACMEQ collected its latest round of data in 14 countries in East and Southern Africa from 2012-2014. These include Botswana, Kenya, Lesotho, Mauritius, Malawi, Mozambique, Namibia, Seychelles, South Africa, Tanzania, Uganda, Zambia, Zanzibar and Zimbabwe. SACMEQ was designed and scaled to be comparable to past rounds. We include microdata from prior rounds, and estimates from reports for the latest round of SACMEQ since the microdata are pending.

*PASEC***.** The "Programme d'Analyse des Systèmes Éducatifs" (PASEC, or "Programme of Analysis of Education Systems") was launched by the Conference of Ministers of Education of French-Speaking Countries (CONFEMEN). These surveys are conducted in French-speaking countries in Sub-Saharan Africa in primary school (grades 2 and 5) in math and French. Each round includes 10 countries. PASEC I occurred from 1996 to 2003; PASEC II from 2004 to 2010 and PASEC III was conducted in 2014. Of note, PASEC has not always been conducted simultaneously across countries and participation has varied considerably since 1994. The following is a list of participating countries before 2014 in chronological order: Djibouti, Congo, Mali, Central African Republic, Senegal, Burkina Faso, Cameroon, Côte d'Ivoire, Madagascar, Guinea, Togo, Niger, Chad, Mauritania, Guinea, Benin, Mauritius, Republic of Congo, Burundi, Comoros, Lebanon, Democratic Republic of Congo. Additional countries took a slightly different test between 2010 and 2011 (Lao PDR, Mali, Cambodia and Vietnam). The most recent PASEC in 2014 uses Item Response Theory (IRT). Ten countries participated, including Benin, Burkina Faso, Burundi, Cameroon, Chad, Republic of Congo, Côte d'Ivoire, Niger, Senegal and Togo. We include these countries using available microdata. Madagascar also participated in 2015 and was scaled to the PASEC 2014 round. We include Madagascar in our database using estimates from reports. To provide a link to past PASEC rounds, which used classical test theory, we create an inter-temporal comparison using a linking function derived based on Togo, which participated in all rounds of PASEC. However, given that PASEC did not conduct intertemporal scaling calibration directly, intertemporal comparisons for PASEC should be analyzed with this caveat in mind.

*LLECE***.** The Latin American Laboratory for Assessment of the Quality of Education (LLECE) was formed in 1994 and is coordinated by the UNESCO Regional Bureau for Education in Latin America and the Caribbean. Assessments conducted by the LLECE focus on achievement in reading and mathematics in primary school. The first round was conducted in 1998 across grades 3 and 4 in 13 countries. These countries include: Argentina, Bolivia, Brazil, Chile, Colombia, Costa Rica, Cuba, Dominican Republic, Honduras, Mexico, Paraguay, Peru and Venezuela. The second round of the LLECE survey was initiated in 2006 in the same countries as LLECE I. In round two, called the Second Regional Comparative and Explanatory Study (SERCE), pupils were tested in grade 3 and grade 6. The Third Regional Comparative and Explanatory Study (TERCE), was done in 2013 across grades 3 and 6 and included 15 Latin American and Caribbean countries. We only include SERCE and TERCE data in this database, since these assessments are most similar and cover comparable grades.

3. The Early Grade Reading Assessment (EGRA)

The Early Grade Reading Assessment (EGRA) is a basic literacy assessment conducted in early grades. The assessment is conducted most often in grades 2-4. Since 2006, EGRA has been conducted in over 65 countries. EGRA was developed by RTI and is typically implemented by USAID, RTI and local partners.[46]

The assessment is a short oral assessment conducted with a child one-on-one. EGRA is designed to be flexible and adapted across countries and contexts, while maintaining core modules and similarities. EGRA is a timed test, enabling uniformity in how it is conducted. The tests often represent the most common features of the local language and align with the expectations of the grade level. EGRA includes up to thirteen subtasks, such as 'oral reading fluency', 'vocabulary', 'diction', and 'reading comprehension.' Multiple questions are included in each subtask to test proficiency. Of the thirteen subtasks, there are a few subtasks encouraged to be delivered across all countries and contexts.[46]

We compile and include data from the 'reading comprehension' indicator in EGRA from 48 countries. This indicator is available in nearly all EGRA data sets and is less sensitive to differences in context, implementation and language. It also and has a strong conceptual link to RSATs and ISATs[47,48] which also measure reading comprehension. To ensure robustness to language effects, we only include data when students took the test in their language of instruction. We use data for grades 2-4, which EGRA is designed for, although certain countries will participate out of this range. We restrict data used for our database to grades 2-4 to be consistent with the design of EGRA. We scale the EGRA microdata to a mean of 500 and standard deviation of 100. This scale corresponds to the scale used by RSATs and ISATs. We include all EGRA data from 2007-2017 as one round. This ensures our scaling is not biased by changing distributions of countries. In the future, we will consider new EGRA data as part of a future round and will conduct intertemporal comparisons using a similar approach to PISA.[4] Patrinos and Angrist (2018) provide additional detailed analysis and robustness checks on the inclusion of EGRA data.[49]

The inclusion of EGRA adds 48 countries to the database with at least one data point in the past 10 years, nearly all of which are developing economies. Of the 48 countries, nearly two-thirds (31 countries) have data that is nationally representative. Linking functions for EGRA are derived

using countries with nationally representative data only, to ensure the assumptions underlying the construction of the linking function hold. We include countries with non-representative data only when the alternative is no data. We include a dummy variable indicating when the data is not nationally representative to enable users of the database to analyze the data accordingly.

4. Summary of Assessments Included in the Database

We include seven learning assessments in our database. Supplement Table 4 summarizes the assessments included. Supplement Table 5 further describes the distribution of source assessments included in our database by country-level-year observations. Most regional assessments are done at the primary level. Moreover, regional assessments comprise nearly 40 percent of primary country-level-year observations, marking substantial representation of developing countries.

Supplement Table 4 | Review of student achievement tests

Organization	Abbr.	Year	Subject	Countries/ Areas	Grade/Age
IEA	TIMSS	Every four years since 2003 (latest round is 2015)	M,S	38, 26, 48, 66, 65	4,8
UNESCO	LLECE	2006, 2013	M,S,R	13, 16 (only 6 for science)	3,6
UNESCO	SACMEQ	2000, 2003, 2007, 2013	M,R	7, 15, 16	6<br>6
CONFEMEN	PASEC	2006, 2014	M,R	22 (before 2014), 10	Until 2014: 2,5<br>After 2014: 3, 6
IEA	PIRLS	Every five years since 2001 (latest round is 2016)	R	35, 41, 55	4
OECD	PISA	Every three years since 2000 (latest round is 2015)	M,S,R	43, 41, 57, 74, 65, 71	Age 15
RTI/USAID	EGRA	2007-2017	R	65	2,3,4

Notes: When denoting subjects, M=math; S=science; and R=reading.

Supplement Table 5 | Distribution of source test for HLO

	Total		Primary		Secondary	
Source Test	Frequency	Percent	Frequency	Percent	Frequency	Percent
EGRA	72	0.04	72	0.10	0	0.00
LLECE	86	0.04	86	0.12	0	0.00
PASEC	30	0.01	30	0.04	0	0.00
PIRLS	160	0.08	160	0.22	0	0.00
PISA	951	0.47	0	0.00	951	0.73
SACMEQ	78	0.04	78	0.11	0	0.00
TIMSS	646	0.32	298	0.41	348	0.27
Total	2023		724		1299	

Notes: We include country-year-level observation counts by source test based on the metadata.

E. Additional Methodological Parameters

Over-time Comparability.— ISATs and RSATs have been designed to be comparable since the late 1990s and early 2000s. Thus, the use of these modern assessments enables comparability over time from this time period onwards.

Time Intervals.— While this is one of the largest and most comprehensive comparable learning outcomes databases produced to date, it is still sparse given limited test frequency. In other databases, the data is often disaggregated over 5-year periods. This produces continuously spaced intervals, is designed to reduce noise by averaging results within these intervals and is comparable to the Barro-Lee approach for years of schooling. In the metadata, we provide the exact year of test as documented in official reports. This enables greater granularity and precision of the data and enables the users of the database to make trade-offs at their discretion.

Schooling Levels.— We construct a score for each grade and pool across grades within each schooling level to produce primary and secondary school scores. We distinguish primary from secondary schooling since enrollment rates drop off between levels in many developing countries. This introduces a potential selection term in secondary school scores, with the highest performing students progressing in the system, biasing scores up due to selection rather than actual learning.

Conceptually, the broader categories of 'primary' and 'secondary' scores enable us to categorize learning at schooling levels across assessments which span multiple grades and age groups. If the test is designed for an age group (for example, PISA) we code it at the relevant schooling level (for example, secondary for PISA). We specify an approach to including specific grade levels to ensure we have a tight grade interval within one to two years to minimize scope for grade-fixed effects. While the interval is relatively small, it still leaves room for grade-fixed effects rather than test-fixed effects when linking tests. For example, linking PIRLS 2001 grade 4 with SACMEQ 2000 grade 6 might capture a grade difference in PIRLS in addition to difficulty. However, to enable greater country coverage, we put up with the need to expand beyond single grade level intervals. Moreover, these differences are often small and since linking functions are applied to all tests being linked, original ranks will be preserved. An analysis of EGRA in Patrinos and Angrist (2018)[49] demonstrates sensitivity to grade. A regression with and without grade-fixed effects comparing mean scores relative to a country which participates across all three grade levels 2-4. shows small differences, with near complete overlap in the confidence intervals on the grade and non-grade-fixed estimates. This sensitivity analysis increases confidence that EGRA data, and other regional assessment data, is robust to data availability by grade.

Subjects.— We construct linking functions specific to reading math, and science. While the proficiency is not granular at the test item level, this ensures that there is significant proficiency overlap when tests are being put on a global scale.

Subsamples.—When calculating the HLO by gender we apply the average linking function to each subsample, rather than constructing subsample specific linking functions. While performance is likely to vary across subsamples in a given test, the relationship between pair-wise tests being linked is unlikely to vary across subsamples nor relative to the full sample.

Metadata.— Our database is disaggregated by subject, schooling level, grade, year and source test. We call this version the 'metadata.' If scores exist for an international standard achievement test (ISAT) already, such as TIMSS in math and science, PIRLS in primary reading, or PISA in secondary reading, we include those scores. If no ISAT scores exist for a given country-year observation, we include the scores generated through linking function. Of note, while we conduct sensitivity tests on all scores derived from all source tests, since PASEC is the least reliable linking function, in particular for math scores, we only include reading scores in the final metadata and analysis.

The data series used in the Human Capital Index (HCI) aggregates the metadata presented in this paper. The aggregation used in the HCI is described in depth in Kraay (2019).[25] There are multiple ways to aggregate the data. For example, the HCI averages data across schooling levels and subjects and uses the most recent year available. The HCI further combines data differently depending on the testing source, for example, including EGRA data in the final time series only when no other data is available. This implicitly weights the importance of testing source over schooling level or subject. Alternative aggregations of the metadata are possible. We present the metadata in this database to enable users to make judgements based on the purpose of their analysis and for maximum transparency.

Analysis Sample.— In the analysis for the paper, we use the underlying metadata, merged with other datasets for each analytical exercise, as described in the main text and methods section. Moreover, while in the metadata we include all nationally representative as well as the non-nationally representative data, in the analysis sample we only include non-nationally representative data if no other data is available for a given country-subject-level observation within a given source test. In both cases, all 164 countries are included, but in the analysis we do not, for example, use data from non-nationally representative EGRAs in Kenya since we have nationally representative SACMEQ data. In the metadata we make all data available and describe the features of each datapoint to enable full transparency and for users to make trade-offs accordingly.

Exceptions.— In unusual cases, the procedures practiced for a given international or regional test are adapted for the country context. Sri Lanka took a national assessment with items linked to the PISA test to provide comparable scores. Sixth grade students in Botswana took TIMSS instead of fourth grade in 2011. Another example includes India and China, where only certain states and cities participated in PISA. These variations are acknowledged by the underlying tests and the data is caveated with an asterisk in published reports. We preserve this information in our data and include notes in the metadata for each case. In the case of India, we verify that the state data is likely to be nationally representative using national assessment data.

We make an adjustment beyond the underlying tests in the case of China given the likelihood that China's current PISA data is biased. The China HLO based on 2015 PISA data is from four cities (Beijing, Shanghai, Jiangsu, and Guangdong) and is 532. However, this data is likely biased upwards since the cities participating are urban and rich relative to the national average. We adjust the score based on socioeconomic information by city and across the nation and produce an average HLO of 462 at the secondary level, which is plausibly representative at the national level. The detailed procedure is described in Patrinos and Angrist (2018).[49] In the metadata we include both original non-nationally representative scores, as well as adjusted representative scores.

Supplemental References

45. Gneezy, Uri, John A. List, Jeffrey A. Livingston, Sally Sadoff, Xiangdong Qin, and Yang Xu. *Measuring success in education: the role of effort on the test itself*. No. w24004. National Bureau of Economic Research, 2017.
46. Gove, Amber. *Early Grade Reading Assessment Toolkit.* RTI International, USAID and the World Bank, 2009.
47. Dubeck, Margaret M. and Amber Gove. "The Early Grade Reading Assessment (EGRA): Its Theoretical Foundation, Purpose, and Limitations." *International Journal of Educational Development* 40 (2015): 315-322.
48. Abadzi, Helen. "Efficient learning for the poor: New insights into literacy acquisition for children." *International Review of Education* 54, no. 5-6 (2008): 581-604.
49. Patrinos, Harry Anthony, and Noam Angrist. *Global Dataset on Education Quality: A Review and Update (2000–2017)*. The World Bank, 2018.

Supplemental Tables

Supplement Table 6 | Test linking architecture

Test X	Test Y	Subject	Level	Overlapping Countries
PISA	TIMSS	Math, Science	Secondary	Australia, Bulgaria, Canada, Chile, Chinese Taipei, Colombia, Czech Republic, Finland, Georgia, Hong Kong – China, Hungary, Indonesia, Israel, Italy, Japan, Jordan, Kazakhstan, Korea, Republic of, Latvia, Lebanon, Lithuania, Macedonia F.Y.R., Malaysia, Malta, Netherlands, New Zealand, Norway, Qatar, Romania, Russian Federation, Serbia, Singapore, Slovakia, Slovenia, Sweden, Thailand, Tunisia, Turkey, USA, UAE.
SACMEQ	PIRLS	Reading	Primary	Botswana
SACMEQ	TIMSS	Math	Primary	Botswana
LLECE	PIRLS	Reading	Primary	Colombia, Chile, Honduras
LLECE	TIMSS	Math, Science	Primary	Colombia, Chile, Honduras, El Salvador
PASEC Round 1	SACMEQ	Reading, Math	Primary	Mauritius
PASEC Round 2	PASEC Round 1	Reading, Math	Primary	Togo
EGRA	PIRLS	Reading	Primary	Egypt, Honduras, Indonesia

Notes: For ease of representation, we include countries used at any point in time for each test linking procedure. In some rounds, some countries are not included, since we specify that for a given round to be linked, tests should be administered in adjacent years. A more detailed architecture by year is available on request.

Supplemental Figures

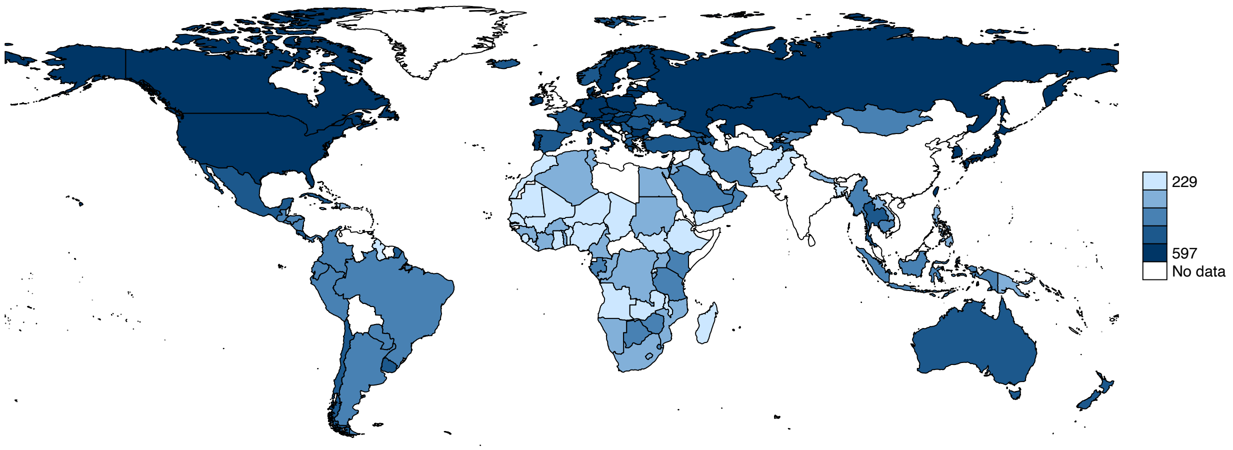

Supplement Figure 9 | Primary learning score

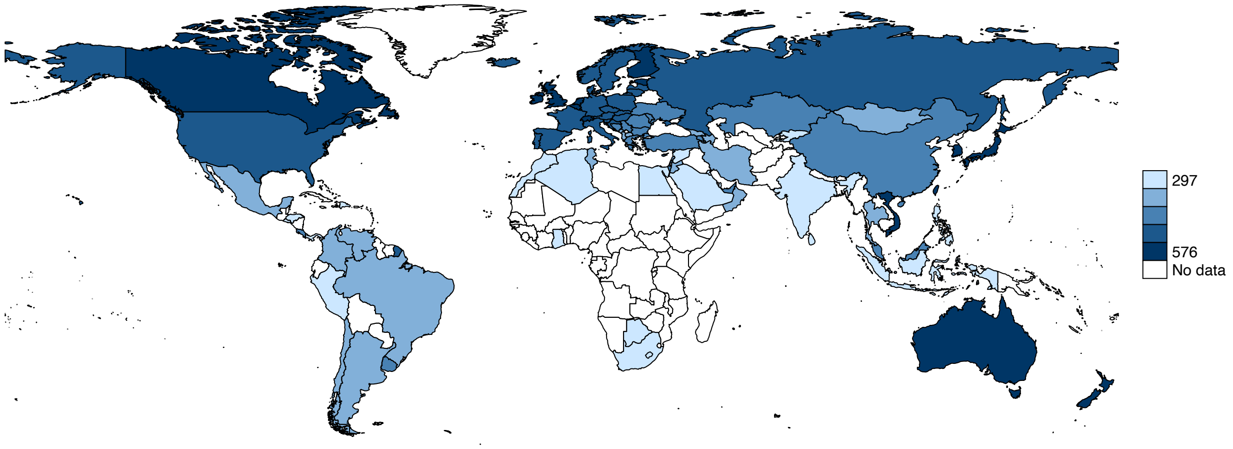

Supplement Figure 10 | Secondary learning score

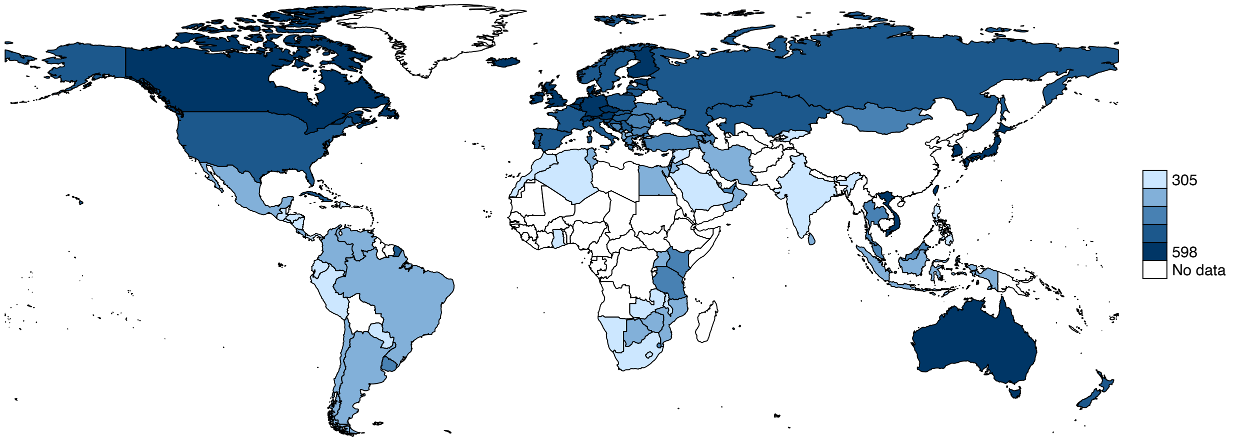

Supplement Figure 11 | Math learning score

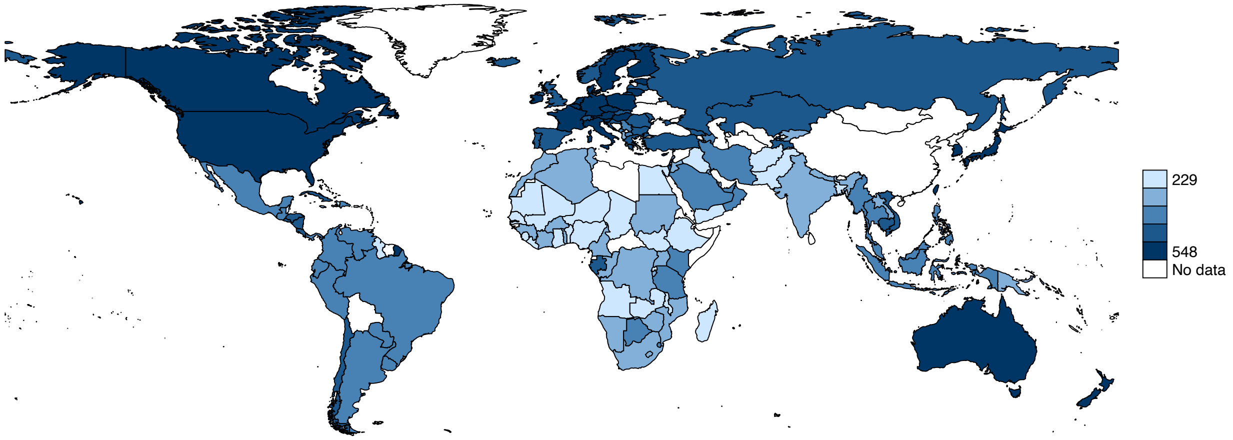

Supplement Figure 12 | Reading learning score

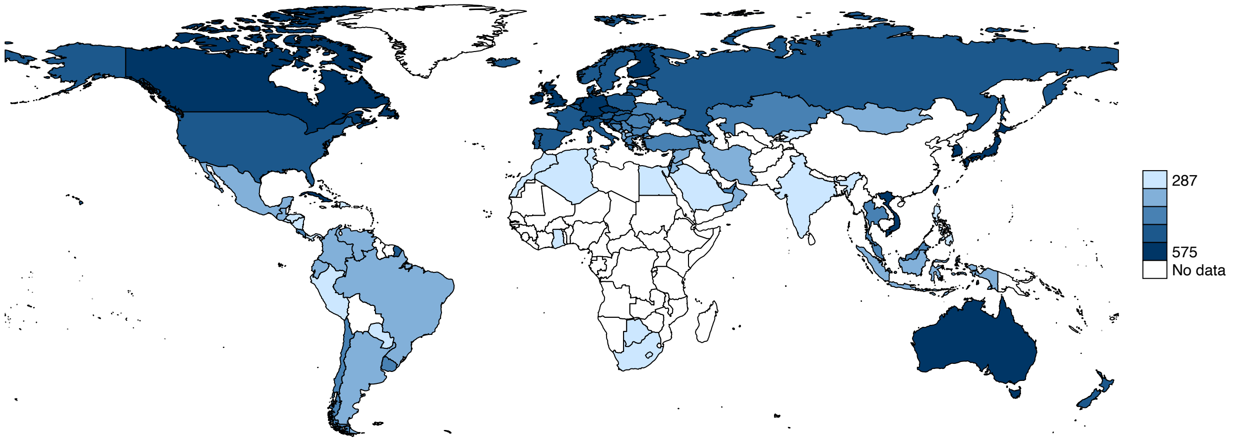

Supplement Figure 13 | Science learning score

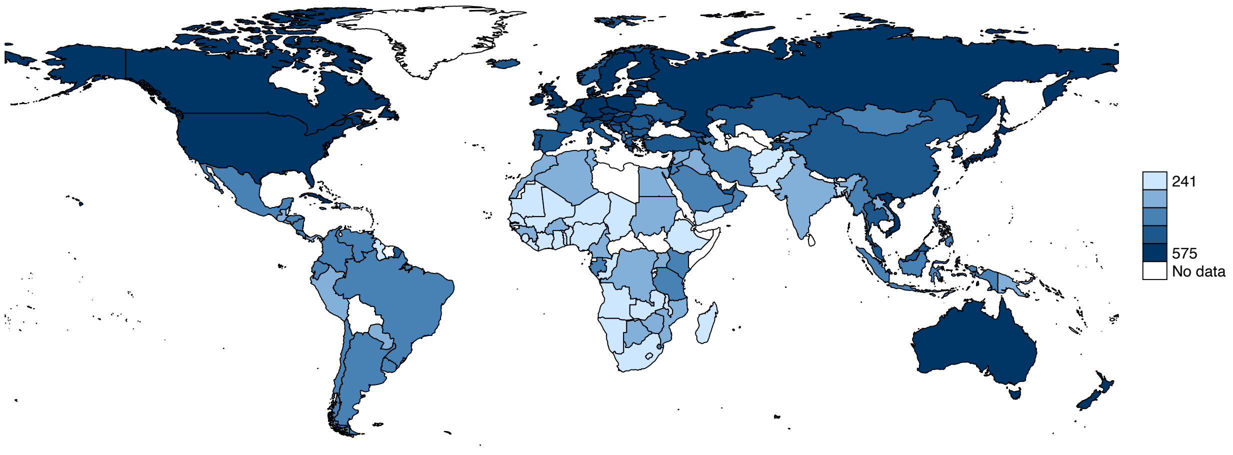

Supplement Figure 14 | Female learning score

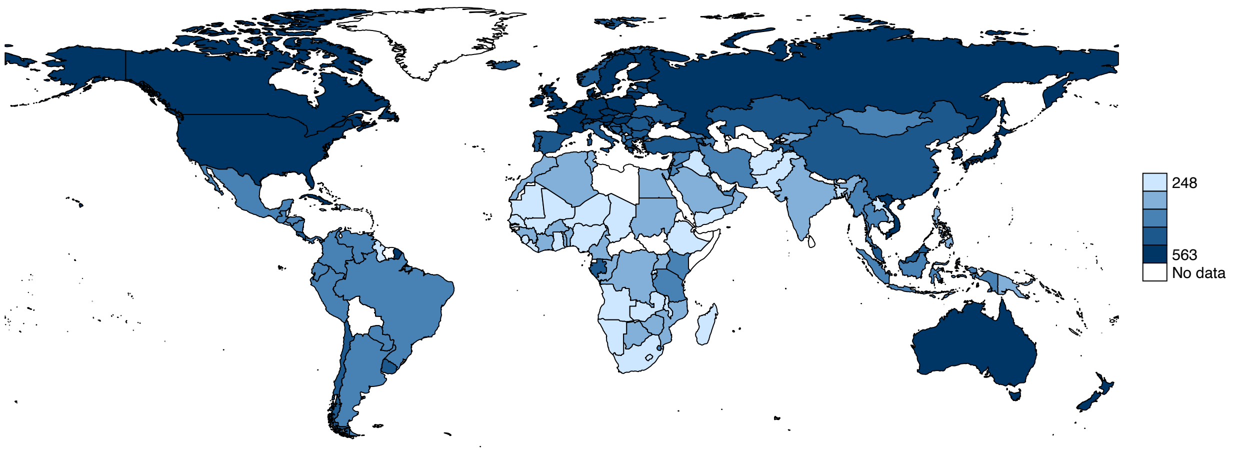

Supplement Figure 15 | Male learning score

Notes: All maps are produced by the authors and do not require a license to be used. Each figure includes average scores from 2000-2017 for a given disaggregation of the data (by gender, by level of schooling, by subject). The legend and blue colored bars denote the average Harmonized Learning Outcome (HLO).
