# Supplementary material for: Measuring human capital using global learning data
Source: Nature. 2021 Mar 10;592(7854):403–8. doi: 10.1038/s41586-021-03323-7 (PMC8046667; doi:10.1038/s41586-021-03323-7)
Supplement: Supplementary file 2 — Reporting Summary [file 41586_2021_3323_MOESM2_ESM.pdf]

nature research

Corresponding author(s): Noam Angrist
Last updated by author(s): Jan 26 2021

# Reporting Summary

Nature Research wishes to improve the reproducibility of the work that we publish. This form provides structure for consistency and transparency in reporting. For further information on Nature Research policies, see our Editorial Policies and the Editorial Policy Checklist.

## Statistics

For all statistical analyses, confirm that the following items are present in the figure legend, table legend, main text, or Methods section.

| n/a | Confirmed | |
|---|---|---|
| ☒ | ☐ | The exact sample size (*n*) for each experimental group/condition, given as a discrete number and unit of measurement |
| ☒ | ☐ | A statement on whether measurements were taken from distinct samples or whether the same sample was measured repeatedly |
| ☐ | ☒ | The statistical test(s) used AND whether they are one- or two-sided<br>*Only common tests should be described solely by name; describe more complex techniques in the Methods section.* |
| ☐ | ☒ | A description of all covariates tested |
| ☐ | ☒ | A description of any assumptions or corrections, such as tests of normality and adjustment for multiple comparisons |
| ☐ | ☒ | A full description of the statistical parameters including central tendency (e.g. means) or other basic estimates (e.g. regression coefficient) AND variation (e.g. standard deviation) or associated estimates of uncertainty (e.g. confidence intervals) |
| ☐ | ☒ | For null hypothesis testing, the test statistic (e.g. *F*, *t*, *r*) with confidence intervals, effect sizes, degrees of freedom and *P* value noted<br>*Give P values as exact values whenever suitable.* |
| ☒ | ☐ | For Bayesian analysis, information on the choice of priors and Markov chain Monte Carlo settings |
| ☒ | ☐ | For hierarchical and complex designs, identification of the appropriate level for tests and full reporting of outcomes |
| ☐ | ☒ | Estimates of effect sizes (e.g. Cohen's *d*, Pearson's *r*), indicating how they were calculated |

*Our web collection on statistics for biologists contains articles on many of the points above.*

## Software and code

Policy information about availability of computer code

| | |
|---|---|
| Data collection | We build code in Stata to replicate original test score data as well as collate and aggregate a global metadata database |
| Data analysis | We analyze data in Stata version 15.1. Code used for analyses in the paper will be made publicly available on github: https://github.com/measuringhumancapital |

For manuscripts utilizing custom algorithms or software that are central to the research but not yet described in published literature, software must be made available to editors and reviewers. We strongly encourage code deposition in a community repository (e.g. GitHub). See the Nature Research guidelines for submitting code & software for further information.

## Data

Policy information about availability of data

All manuscripts must include a data availability statement. This statement should provide the following information, where applicable:

- Accession codes, unique identifiers, or web links for publicly available datasets
- A list of figures that have associated raw data
- A description of any restrictions on data availability

The data will be made available for public use and updated regularly on the World Bank microdata catalog: https://microdata.worldbank.org. The database is expected to be updated every 2-3 years as new learning data becomes available. The database will be updated at the same location on the microdata World Bank website using the methodology and approach in this paper, with accompanying technical notes on additional countries and data sources added. This study used a combination of data sources, including data that are available from online repositories and required straightforward registration and usage agreement (PISA, TIMSS, PIRLS, SACMEQ, PASEC, LLECE). We also collected data from over 48 countries directly in collaboration with the World Bank and USAID for previously conducted EGRA assessments. All maps presented in this study have been produced by the authors and no permissions are required for publication.

# Field-specific reporting

Please select the one below that is the best fit for your research. If you are not sure, read the appropriate sections before making your selection.

☐ Life sciences ☒ Behavioural & social sciences ☐ Ecological, evolutionary & environmental sciences

For a reference copy of the document with all sections, see nature.com/documents/nr-reporting-summary-flat.pdf

# Behavioural & social sciences study design

All studies must disclose on these points even when the disclosure is negative.

| | |
|---|---|
| Study description | Quantitative aggregation of global learning data from 164 countries and quantitative analysis. |
| Research sample | Learning outcomes for 164 countries where learning outcomes data is available. Data is disaggregated by gender, schooling level (primary versus secondary) and subject (math, reading and science). Data is denoted by the source test and whether the data is nationally representative (which is the case for 91% of countries). Extended Data Table 1 and Supplement Table 1 include additional details. |
| Sampling strategy | Random sample and some stratified with survey weights based on source data available. Source tests typically conduct surveys which are nationally representative and include thousands of participants. Sampling procedures are outlined for each source tests in their respective technical reports. For smaller countries, such as pacific islands, some tests are a census, covering all students in a given grade. |
| Data collection | Most tests are school-based paper tests (PISA, TIMSS, PIRLS, SACMEQ, PASEC, LLECE). EGRA is conducted through one-one-one oral assessment. Supplement Table 4 describes how frequently each test is conducted and the administering agency. |
| Timing | We include data from 2000-2017 |
| Data exclusions | All source test data was used. |
| Non-participation | N/A |
| Randomization | No experimental groups |

# Reporting for specific materials, systems and methods

We require information from authors about some types of materials, experimental systems and methods used in many studies. Here, indicate whether each material, system or method listed is relevant to your study. If you are not sure if a list item applies to your research, read the appropriate section before selecting a response.

## Materials & experimental systems

| n/a | Involved in the study | |
|---|---|---|
| ☒ | ☐ | Antibodies |
| ☒ | ☐ | Eukaryotic cell lines |
| ☒ | ☐ | Palaeontology and archaeology |
| ☒ | ☐ | Animals and other organisms |
| ☐ | ☒ | Human research participants |
| ☒ | ☐ | Clinical data |
| ☒ | ☐ | Dual use research of concern |

## Methods

| n/a | Involved in the study | |
|---|---|---|
| ☒ | ☐ | ChIP-seq |
| ☒ | ☐ | Flow cytometry |
| ☒ | ☐ | MRI-based neuroimaging |

## Human research participants

Policy information about studies involving human research participants

| | |
|---|---|
| Population characteristics | Students in primary and junior school. |
| Recruitment | This paper conducts secondary data analysis only. Primary data collection, recruitment, and consent was done by source test agencies (PISA, TIMSS, PIRLS, SACMEQ, PASEC, LLECE, EGRA) and is described in depth in their respective technical reports. |
| Ethics oversight | This paper conducts secondary data analysis only. Primary data collection, recruitment, and consent was done by source test agencies (PISA, TIMSS, PIRLS, SACMEQ, PASEC, LLECE, EGRA) and is described in depth in their respective technical reports. |

Note that full information on the approval of the study protocol must also be provided in the manuscript.
